# Bayesian reconstruction and differential testing of excised introns

**DOI:** 10.1093/bioinformatics/btaf646

**Published:** 2025-12-01

**Authors:** Marjan Hosseini, Devin McConnell, Derek Aguiar

**Affiliations:** School of Computing, University of Connecticut, Storrs, CT 06269, United States; School of Computing, University of Connecticut, Storrs, CT 06269, United States; School of Computing, University of Connecticut, Storrs, CT 06269, United States; Institute for Systems Genomics, University of Connecticut, Storrs, CT 06269, United States

## Abstract

**Motivation:**

Characterizing the differential excision of introns is critical for understanding the functional complexity of a cell or tissue, from normal developmental processes to disease pathogenesis. Most transcript reconstruction methods infer *full-length transcripts* from high-throughput sequencing data. However, this is a challenging task due to incomplete annotations and the heterogeneous expression of transcripts across cell-types, tissues, and experimental conditions. Several recent methods circumvent these difficulties by considering *local splicing events*, but these methods lose transcript-level splicing information and may conflate similar, but distinct transcripts.

**Results:**

In this work, we formalize a new transcript reconstruction problem that interpolates between the full-length and local splicing perspectives by considering sequences of exon–exon junctions (SEEJs) that co-occur in transcripts. We then present a hierarchical Bayesian admixture model and posterior inference algorithms for computing SEEJs (BSEEJ), and a generalized linear model for characterizing differential SEEJ usage based on model parameter estimates. We show that BSEEJ achieves high $F_{1}$ score for reconstruction tasks and improved accuracy and sensitivity in differential splicing when compared with six transcript and local splicing methods on simulated data. Lastly, we evaluate BSEEJ on experimental data based on transcript reconstruction, novelty of transcripts produced, model sensitivity to hyperparameters, and a functional analysis of differentially expressed SEEJs.

**Availability and implementation:**

BSEEJ is freely available at https://github.com/bayesomicslab/BSEEJ.

## Graphical abstract

**Figure:**
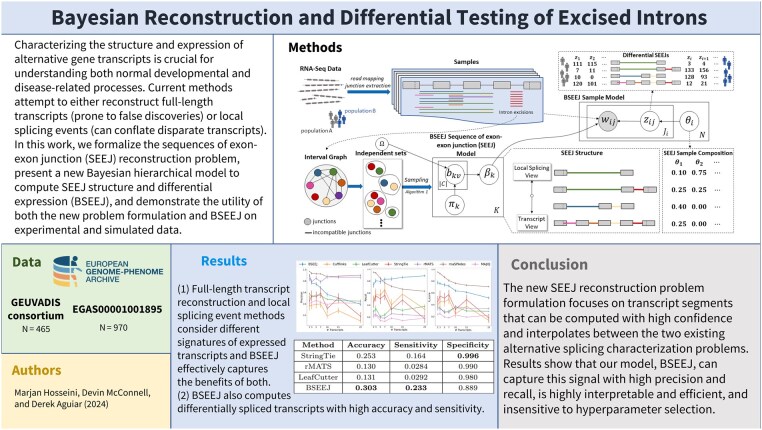

## 1 Introduction

The alternative splicing of exons enables a single gene to produce multiple distinct and functionally diverse protein isoforms (Wilkinson *et al.* 2020). Alternative splicing is prevalent, affecting 95% of human multi-exon genes (Pan *et al.* 2008), and is crucial for human adaptation (Barbosa-Morais *et al.* 2012), gene regulation, tissue identity (Barbosa-Morais *et al.* 2012, Baralle and Giudice 2017), disease etiology, and drug resistance (Lee and Abdel-Wahab 2016, Yang *et al.* 2019). High-throughput RNA sequencing (RNA-seq) facilitates the discovery and quantification of alternative splicing from short-read data. After sequencing, reads are mapped to a reference genome with a splice-aware aligner for transcript discovery and quantification (Dobin *et al.* 2013). Reads mapping to regions where intronic RNA was removed (i.e. *splice junctions*) are informative of the latent transcript structure and can be assembled into transcripts *de novo* or using reference annotations.

Characterizing alternative splicing computationally is challenging due to biological variability and technological limitations. Transcript structures and frequencies vary by population, sex, tissue, and cell type (Park *et al.* 2018, GTEx Consortium 2020). Many cell-types, cell states, or non-model organisms have unknown or incomplete transcriptomes (Morillon and Gautheret 2019). Distinguishing the transcript of origin for a read is difficult since distinct alternatively spliced transcripts from the same gene may have significant sequence overlaps. Lastly, the number of splice junctions in a single read is limited in short-read sequencing; long-read sequencing produces observations with more junctions, but is costly, error-prone, and lower throughput (Mantere *et al.* 2019).

Despite these challenges, many transcript reconstruction and quantification methods exist, primarily focused on reconstructing *full-length* transcripts defined by their composite exons (Trapnell *et al.* 2010, Pertea *et al.* 2015, Vaquero-Garcia *et al.* 2016, Aguiar *et al.* 2018, Bushmanova *et al.* 2019, Shi *et al.* 2024). Cufflinks, StringTie, and rnaSPAdes are well-established graph-based methods that construct full-length transcripts, with or without annotations. Cufflinks reconstructs transcripts as minimum paths in an associated graph, where the aligned reads are vertices, and edges denote the compatibility of isoforms (Trapnell *et al.* 2010). StringTie models transcript reconstruction using maximum network flow on a splice graph, where paths and read coverage inform isoform composition and quantification respectively (Pertea *et al.* 2015). The *de novo* transcript reconstruction method rnaSPAdes is based on a modified de Bruijn graph approach, and can be combined with Salmon (Patro *et al.* 2015) for transcript abundance quantification (Bushmanova *et al.* 2019). However, the transcript reconstruction problem is underdetermined for most genes and variability of read depths due to technical artifacts or biological biases obfuscates reconstruction and quantification (McIntyre *et al.* 2011). In fact, full-length transcripts can be difficult to reconstruct and quantify even when transcriptome annotations are known (Vaquero-Garcia *et al.* 2016).

**Figure 1.**
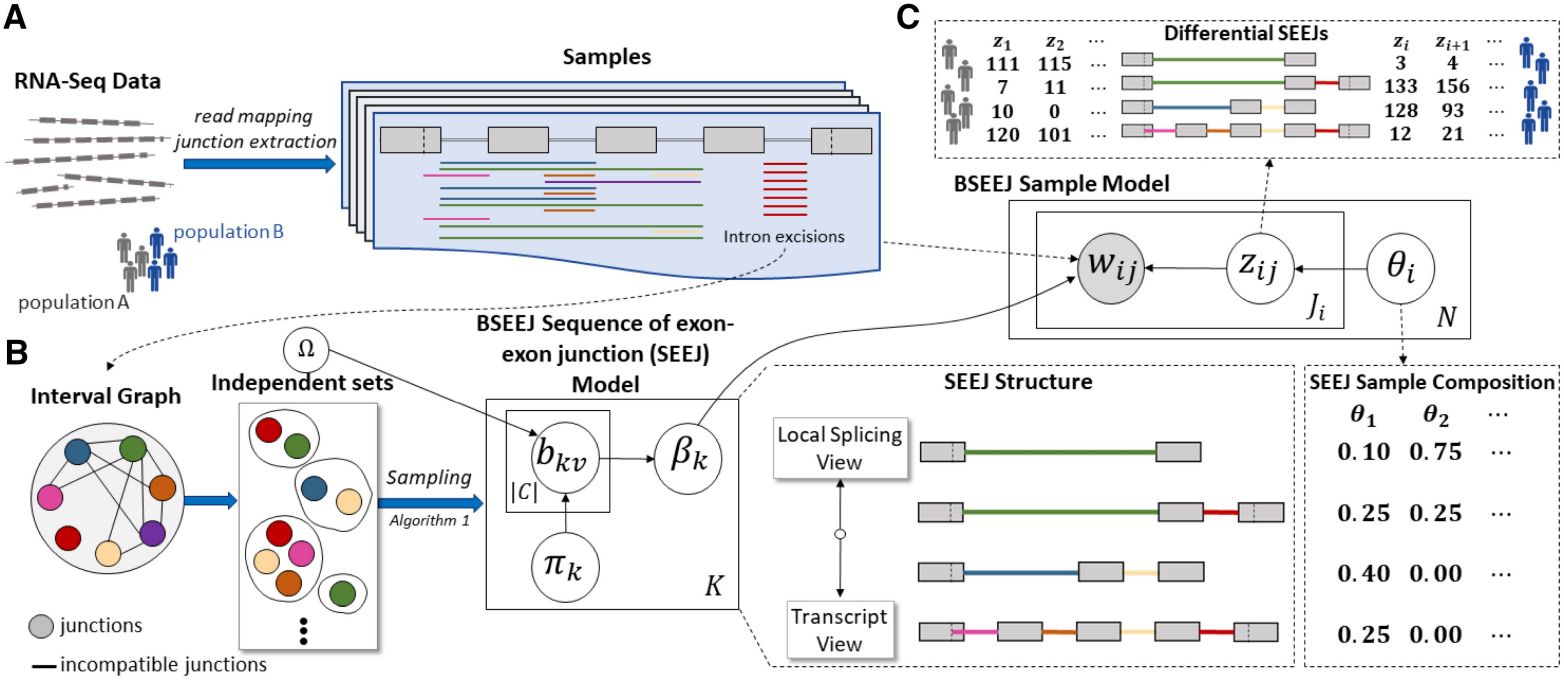
BSEEJ overview. (A) Junctions are extracted from RNA-seq data and (B) used to construct a graph where nodes are junctions and edges connect two junctions if they overlap. BSEEJ is a Bayesian admixture model that reconstructs sequences of exon-exon junctions (SEEJs), which are informed by incompatibilities encoded in an interval graph. (C) Posterior inference algorithms yield SEEJs and reads mapped to SEEJs, which are used to compute differential SEEJ usage. An example of how local splicing methods can conflate transcripts is given by the green junction. Full-length methods may struggle differentiating the last two transcripts due to their large sequence overlap. In both cases, these issues may affect differential splicing estimates.

In contrast, *local* splicing methods assess singular splicing events where an intron is excised during the production of mature mRNA. A splicing event can be represented as a sequence of exon-exon junctions (or simply, junctions), which are intervals spanning from the starting and terminal positions of the excised introns. The local splicing method rMATS detects differential exon usage in reads that overlap junctions (i.e. *junction reads*) among five alternative splicing events (Shen *et al.* 2014). In contrast, LeafCutter models excised introns rather than the constituent exons by constructing graphs where edges connect junctions sharing a donor or acceptor splice site (Li *et al.* 2018). Since junctions are easier to compute than transcripts, *local* splicing methods are ideal for detecting differential splicing events in low coverage data or for samples with incomplete annotations (Li *et al.* 2018); but, these methods are limited to small neighborhoods around a splicing event (Shen *et al.* 2014, Vaquero-Garcia *et al.* 2016, 2023, Li *et al.* 2018, Mertes *et al.* 2021) and thus may conflate transcripts that share junctions (Fig. 1), making (i) quantification and downstream haplotype analysis difficult, and (ii) differential expression subject to ambiguity of transcript-level contributions. Additional related work can be found in the Supplementary Materials (Supplementary Section Related Work, available as supplementary data at *Bioinformatics* online).

Here, we formalize the *SEEJ reconstruction problem*, a new problem formulation for transcript reconstruction focusing on variable-length sequences of exon–exon junctions (SEEJs). Then, we develop a hierarchical **B**ayesian admixture model for computing **SEEJ**s (BSEEJ) and a generalized linear model (GLM) for characterizing differential SEEJ usage based on model parameter estimates (Fig. 1); admixture refers to samples being modeled as collections of junction reads that are themselves sampled from global mixture components (SEEJs). BSEEJ jointly considers all samples in a formal probabilistic model, does not require exon or transcript annotations, and enables *local*-to-*full-length* transcript reconstruction. This middle ground approach is suitable in complex genomic loci such as the human leukocyte antigen region (Lappalainen *et al.* 2013, Robinson *et al.* 2015). We demonstrate the theoretical compactness of BSEEJ and develop Gibbs sampling and local search-based inference algorithms that model the discovery of new SEEJs as computing independent sets in an interval graph. Results show that BSEEJ achieves high precision, recall, and $F_{1}$ score for transcript reconstruction on simulated data and accurate differential SEEJ identification. Lastly, we evaluate BSEEJ on two experimental datasets based on transcript reconstruction, novelty of produced transcripts, hyperparameter sensitivity, and functional analysis of differentially expressed SEEJs, showing that BSEEJ captures functionally relevant biological signals.

## 2 Materials and methods

Let D be the collection of junction reads for *N* samples (i∈{1,…,N}) extracted from RNA-seq data aligned to a reference genome. Though a challenging problem, we assume aligned sequence reads are assigned to a specific gene for ease of exposition. The **transcript reconstruction problem** aims to reconstruct full-length transcripts as defined by their component exons. In contrast, the **splice event detection problem** aims to identify splicing events that exist in any transcript expressed in D. Since transcript reconstruction is not required, splice event detection is a simpler computational task, but still yields biologically interesting insights, e.g. differentially spliced events.

We introduce the **SEEJ reconstruction problem**: given aligned RNA-seq data D, reconstruct SEEJs that co-occur in expressed transcripts. The problem interpolates between the full-length transcript reconstruction and splice event detection problems, which are special cases of SEEJ reconstruction. Whenever the context is clear, we will refer to both differential usage of local splicing events, transcripts, and SEEJs simply as *differential expression*.

### 2.1 The BSEEJ model

BSEEJ solves the SEEJ reconstruction problem by representing samples as mixtures of SEEJs sampled from a global distribution, i.e. learned across samples (Fig. 1 and Fig. 1, available as supplementary data at *Bioinformatics* online). Briefly, BSEEJ models samples as mixtures of SEEJs, which are themselves collections of junction reads. For each gene, BSEEJ learns the structure of SEEJs, a global distribution over SEEJs, a mapping between junctions reads and SEEJs, and a sample-specific distribution of SEEJs. Let the number of junction reads for sample *i* be $J_{i}$ and the set of unique junctions within a gene be *V*, indexed by v∈{1,…,|V|}. BSEEJ consists of two major components: (a) combinatorial model for SEEJ structure and (b) a probabilistic model for SEEJ admixture. Additional model details can be found in Supplementary Section Additional Model Details, available as supplementary data at *Bioinformatics* online.

#### 2.1.1 Combinatorial model for SEEJ structure

Junctions are formally defined as genomic intervals $\left[\hat{s},\hat{t}\right]$, where $\hat{s}$ and $\hat{t}$ denote the starting and terminal positions of excised introns, respectively. The goal is to arrange junctions into *K* SEEJs while ensuring that no SEEJ contains two overlapping junctions. To enforce this criteria, we generate a graph $G=(V_{g},E_{g})$, where each vertex $\upsilon \in V_{g}$ represents a unique junction and $(\upsilon ,\upsilon^{\prime })\in E_{g}$ if $\upsilon =[\hat{s},\hat{t}]$ and $\upsilon^{\prime }=[{\hat{s}}^{\prime },{\hat{t}}^{\prime }]$ intersect, i.e. $\min(\hat{t},{\hat{t}}^{\prime })-\max(\hat{s},{\hat{s}}^{\prime })>0$. An independent set in *G* corresponds to a valid SEEJ, since no pair of junctions overlap (Fig. 1B).

In BSEEJ, Bernoulli random variables disallow conflicting junction assignments to the same SEEJ. Without loss of generality, we assume that $(\upsilon ,\upsilon^{\prime })\in E_{g}$ and create a Bernoulli random variable $b_{kv}$, where $b_{kv}=1$ if *v* is in the $k^{th}$ SEEJ and 0 otherwise. Enforcing $b_{kv}⊕b_{kv^{\prime }}$ (⊕ is the exclusive OR operator) by creating a *b* variable for each edge in *G* scales poorly since the number of model variables would be proportional to the number of conflicts. For example, the star graph $S_{k}$ has a single internal node and *k* leaves, which would generate *k* Bernoulli random variables. However, if the internal node is selected, only a single Bernoulli variable is required since selecting the junction represented by the central node precludes the selection of any leaf node junction. A parsimonious representation of junction conflicts reduces the number of model parameters, making inference more efficient and mitigating issues associated with non-identifiability. Let *C* be the set of Bernoulli random variables required to encode all conflicts in *G*.

Proposition 1. *Estimating the minimum number of variables required to encode all conflicts between junctions, i.e. computing C s.t.* |*C*| *is minimum, can be done in* $O(|E_{g}|)$.

*Proof.* Since each edge in $E_{g}$ denotes two junctions that cannot coexist in the same SEEJ, we need at least one incident node of each edge to exist in *C*; this is a node cover of *G*. A minimum node cover has the smallest cardinality among all node covers and therefore a corresponding *C* for which |*C*| is the smallest. Since *G* is an interval graph, computation of a minimum node cover can be done in $O(|E_{g}|)$ time (Marathe *et al.* 1992).■

#### 2.1.2 Probabilistic model for SEEJs

The probabilistic component of BSEEJ models SEEJ structure across all samples (Fig. 1B) and SEEJ composition for each sample (Fig. 1C). We place a beta-Bernoulli prior on junctions to control SEEJ sparsity:


$$\begin{array}{c} b_{kv}∼\text{Bernoulli}(\pi_{k}),\forall v\in C,\;s.t.\;\;\;\;b_{k\cdot }\in \Omega \\ \pi_{k}∼\text{Beta}(r,s), \end{array}$$

for hyperparameters *r* and *s* and the space of valid SEEJs Ω, which is defined as all valid SEEJs (no junction conflicts); thus, Ω is equivalent to the *set of all (not necessarily maximal) independent sets in G*. In total, we instantiate |*C*| Bernoulli variables since we can encode all v∈C using $b_{kv}$ and all $v^{\prime }\notin C$ with variables of the form $\left(1-b_{kv}\right)$. If a single $v^{\prime }\notin C$ is adjacent to two or more v∈C, one adjacent *v* is selected at random for the encoding. Hyperparameters *r* and *s* can be adjusted to encourage shorter (e.g. splice events) or longer (e.g. transcripts) SEEJs.

We model the *k*th SEEJ as a degenerate Dirichlet distribution whose dimension is controlled by the beta-Bernoulli prior (Aguiar *et al.* 2018): $\beta_{k}∼{\text{Dirichlet}}_{\left|V\right|}(\eta ⊙b_{k})$, where $\eta =(\eta_{1},\ldots ,\eta_{\left|V\right|})$ is a hyperparameter and notation ⊙ denotes element-wise vector multiplication. Non-overlapping junction constraints are modeled in the |*V*|-dimensional $b_{k}=(b_{k1},\ldots ,b_{k|V|})$ vector, in which $b_{kv}$ selectively turns off or on dimension *v*. Note that there are only |*C*| unique $b_{kv}$ variables as some of these variables are repeated due to junction constraints. The equation for $\beta_{k}$ highlights non-identifiability issues when |*C*| is not minimum. For example, consider a simple graph $G=(V_{g},E_{g})$ with two conflicting junctions in edge $E_{g}=\{(\upsilon_{1},\upsilon_{2})\}$. Clearly, both junctions cannot simultaneously belong to the same SEEJ, making $b_{kv_{1}}=b_{kv_{2}}=1$ invalid and modeled by the fact that the Dirichlet distribution will necessarily set both dimensions to 0. Likewise, setting $b_{kv_{1}}=0$ and $b_{kv_{2}}=0$ will also force both dimensions of the Dirichlet to 0. Thus, introducing two variables, $C=\{b_{kv_{1}},b_{kv_{2}}\}$, instead of one, creates redundancy and non-identifiability.

The SEEJ composition of a sample is modeled by a distribution over SEEJs and a mapping between junction reads and SEEJs. The proportion of SEEJs in sample *i* is given by $\theta_{i}∼{\text{Dirichlet}}_{K}(\alpha )$, where the *k*th dimension represents the probability of observing the *k*th SEEJ; $\alpha =(\alpha_{1},\ldots ,\alpha_{K})$ are hyperparameters. The assignment of junction read *j* in sample *i* to a SEEJ is denoted by $z_{ij}\in \{1,\ldots ,K\}$ and follows a multinomial distribution: $z_{ij}∼\text{Multinomial}(\theta_{i})$. The data likelihood for junction read *j* in sample *i* is given by: $w_{ij}∼\text{Multinomial}(\beta_{z_{ij}})$. Here, the parameter for the multinomial is the β selected by variable $z_{ij}$.

### 2.2 Inference algorithm

We fit BSEEJ using Gibbs sampling, which iteratively samples each parameter from their complete conditional distributions (Algorithm 1, derivations can be found in Supplementary Section Inference—Gibbs Sampling, available as supplementary data at *Bioinformatics* online). Most complete conditional distributions leverage conjugacy, but variables *z* and *b* require special considerations.

Algorithm 1. BSEEJ inference algorithm.

**Input:** hyperparameters α, η, *r*, and *s*, and # iterations τ

**Output:** samples of $z_{ij}$, $\theta_{i}$, $\pi_{k}$, $b_{kv}$, $\beta_{k}$

1: Randomly initialize $z_{ij}^{0},\theta_{i}^{0},\pi_{k}^{0},b_{kv}^{0},\beta_{k}^{0}$

2: t←1

3: **for**  t=1 to τ  **do** 

4:   Sample $z_{ij}^{t}∼p(w_{ij}|z_{ij}^{t-1},\beta_{1:K}{\;}^{t-1})p(z_{ij}^{t-1}|\theta_{i}^{t-1})$

5:   Sample $\theta_{i}^{t}∼\prod_{j=1}^{J_{i}}p(z_{ij}^{t}|\theta_{i}^{t-1})p(\theta_{i}^{t-1}|\alpha )$

6:   $m_{k}\leftarrow \sum_{v\in V}1[b_{kv}^{t-1}=1]$

7:   Sample $\pi_{k}^{t}∼\text{Beta}(r+m_{k},s+|V|-m_{k})$

8:   Sample $b_{kv}^{t}∼p(b_{kv}^{t-1}=1|\beta_{k}^{t-1},\pi_{k}^{t},b_{k.}{\;}^{t-1})$

9:   Sample $\beta_{k}^{t}∼p(w_{..}|z_{..}^{t},\beta_{k}^{t-1})p(\beta_{k}^{t-1}|b_{k.}^{t},\eta )$

10:  t←t+1

11: **end for** 

In admixture modeling, naïve sampling of z requires iterating over each data observation. Since the dimensionality of the data is typically much larger than the number of distinct data items in a sample, this causes inference to be inefficient. For example, in topic modeling, the size of the vocabulary typically exceeds the number of unique words in a document. However, in our context, the number of distinct junctions in a gene is less than the number of sequence reads and observations for the same junction are exchangeable; i.e. we consider two distinct junction reads as identical if they overlap the same junctions. Thus, we can compute a single sample from a multinomial distribution instead of *D* categorical samples giving a per-iteration z sampling complexity of O(K+D log(K)), compared to O(DK+D log(K)) for naïve sampling.

Sampling $b_{k}$ is difficult since the distribution of $b_{k}$ is defined over independent sets of *G*. At each iteration we generate 10 new valid SEEJs (independent sets of *G*, not necessarily maximal) ${\left\{\phi_{k\ell }^{t}\right\}}_{\ell =1}^{10}$ using $b_{k}{\;}^{t}$ and $\beta_{k}^{t}$. We generate a $\phi_{k\ell }^{t}$ starting from $b_{k}$. We sample $q∼\text{Bernoulli}(1-\frac{\sum_{v\in V}1[b_{kv}^{t-1}=1]}{\hat{\alpha }(G)})$, where $\hat{\alpha }(G)$ is the size of the maximum independent set of *G*. If q=1, we sample a junction *v* to add to the current SEEJ with probability proportional to $\beta_{kv}$; we only consider junctions that do not conflict with junctions in the current SEEJ and whose $b_{kv}=0$. If q=0, we sample a junction *v* to remove from the current SEEJ with probability proportional to $1-\beta_{kv}$; we only consider junctions in the current SEEJ whose $b_{kv}=1$. Finally, we sample a $\phi_{k\ell }^{t}$ from its complete conditional:


p(ϕkℓt|·)∝πkr+|ϕkℓt|−1(1−πk)s+|N(ϕkℓt)|−1 Γ(∑i∈V∖N(ϕkℓt)ηibki)∏i∈V∖N(ϕkℓt)Γ(ηibki)∏i∈V∖N(ϕkℓt)βkiηibki−1

where $N(\phi_{k\ell }^{t})$ is the neighborhood (adjacent junctions) of $\phi_{k\ell }^{t}$ in *G*. The sampling of $\phi_{k\ell }^{t}$ includes a local independent set search algorithm to enforce junction constraints (Algorithm S2, available as supplementary data at *Bioinformatics* online). We use $\beta_{k}$ from the previous iteration to guide the proposed SEEJs, since $\beta_{k}$ represents junction membership probabilities. After BSEEJ converges, SEEJs with the same configuration are combined. The full Gibbs sampling algorithm derivation can be found in Supplementary Section Inference—Gibbs Sampling, available as supplementary data at *Bioinformatics* online.

### 2.3 Differential SEEJs

For each gene, we quantify differential SEEJ usage across two sample groups based on the expression profile of all SEEJs. Let the vector of mappings from all unique junction read to SEEJs for sample *i* be denoted $z_{i}$. Then, we can express $z_{i}$ as a Dirichlet-multinomial GLM:


$$z_{i1},\ldots ,z_{iJ_{i}}∼\text{DirMult}\left(\sum_{j}z_{ij},\alpha ⊙p_{i}\right).$$

where $p_{ij}=\frac{\;\text{exp}(x_{i}{\hat{\beta }}_{j}+\mu_{j})}{\sum_{k}\;\text{exp}\;(x_{i}{\hat{\beta }}_{k}+\mu_{k})}$ for regression coefficients $\hat{\beta }$ and intercepts μ. We set $\alpha ∼\gamma (1+10^{-4},10^{-4})$ to stabilize maximum likelihood estimation (Li *et al.* 2018). Finally, to test differential SEEJs between two groups, we construct two models: (i) a DirMult GLM where we set $x_{i}=0$ for one group and $x_{i}=1$ for the other and (ii) a DirMult GLM where all $x_{i}=0$. Differential SEEJs are quantified by a likelihood ratio test with k−1 degrees of freedom, where *k* is the number of SEEJs. Note that we assume a common set of SEEJs inferred jointly from all samples.

## 3 Results

We evaluated BSEEJ and three splice event detection methods [rMATS (Shen *et al.* 2014), LeafCutter (Li *et al.* 2018), and MAJIQ (Vaquero-Garcia *et al.* 2023)] and three transcript reconstruction methods [Cufflinks (Trapnell *et al.* 2010), StringTie (Pertea *et al.* 2015), and rnaSPAdes (Bushmanova *et al.* 2019)]. These six methods range from single splicing event to full-length transcripts and so we refer to their reconstructed output collectively as *transcript segments*. SEEJs can be interpreted as the sequence of intron excisions within a transcript segment. Benchmarking these methods is challenging since each approach has a different target for reconstruction. Since splice event detection methods cannot estimate traditional transcript abundance measures (e.g. FPKM), we evaluated each method based on differential splicing detection. With respect to transcript segment reconstruction, measures of accuracy must account for (1) the difference in task difficulty (reconstructing transcripts is more difficult than identifying splicing events) and (2) minor differences in junction starting or terminal positions. Before assessing reconstruction and differential splicing, transcript segments must be aligned to a reference transcriptome.

### 3.1 Evaluation criteria

We represent transcript segments by their junctions. We denote the *v*th junction as $e_{v}$ and define the *k*th transcript segment (or SEEJ) as a subset of junctions, $T_{k}\subseteq \{e_{1},\ldots ,e_{\left|V\right|}\}$. The set of reference transcripts is denoted $T^{r}$ and can either be simulated or experimentally defined based on a well-characterized reference transcriptome. The *partial homogeneity score (phs)* for transcript $T_{k}$ in sample *i* enforces that junctions are sampled from the same reference transcript and is normalized by the size of the computed transcript segment: $s_{i}^{\mathrm{phs}}(T_{k})=\max_{T\in T^{r}}\frac{\sum_{e_{v}\in T_{k}}\;1\left[e_{v}\in T\right]}{\left|T_{k}\right|}$, where $1[e_{v}\in T]$ is 1 if the donor and acceptor splice sites of $e_{v}$ are at most 6 bases from the donor and acceptor splice sites of a junction in *T* (and 0 otherwise).

We also define ${\hat{s}}_{i}^{\mathrm{phs}}$, which normalizes computed transcript segments by the reference transcript length: ${\hat{s}}_{i}^{\mathrm{phs}}(T_{k})=\max_{T\in T^{r}}\frac{\sum_{e_{v}\in T_{k}}\;1\left[e_{v}\in T\right]}{\left|T\right|}$. Both scores $s_{i}^{\mathrm{phs}}$ and ${\hat{s}}_{i}^{\mathrm{phs}}$ are related to the Jaccard index but emphasize different goals. Methods that compute shorter transcript segments will tend to perform better with respect to $s_{i}^{\mathrm{phs}}$ since it is typically easier to produce accurate short transcript segments than full-length transcripts. In contrast, ${\hat{s}}_{i}^{\mathrm{phs}}$ favors longer transcript segments and will be close to 1 if the computed transcript is both accurate and full-length. Either $s_{i}^{\mathrm{phs}}$, ${\hat{s}}_{i}^{\mathrm{phs}}$, Jaccard index, or some linear combination thereof can be used depending on the goals of the study. Finally, an average score for sample *i* can be computed from the set of computed transcripts $T_{\left(i\right)}^{c}=\{T_{k}\}$: $s_{i}^{\mathrm{phs}}\left(T_{\left(i\right)}^{c}\right)=\frac{\sum_{T_{k}\in T_{\left(i\right)}^{c}}s_{i}^{\mathrm{phs}}(T_{k})}{\left|T_{\left(i\right)}^{c}\right|}$ and ${\hat{s}}_{i}^{\mathrm{phs}}\left(T_{\left(i\right)}^{c}\right)=\frac{\sum_{T_{k}\in T_{\left(i\right)}^{c}}{\hat{s}}_{i}^{\mathrm{phs}}(T_{k})}{\left|T_{\left(i\right)}^{c}\right|}$.

To compute precision and recall, we first matched the computed transcript segment $T_{k}$ to the reference transcript $T\in T^{r}$ with maximum $s_{i}^{\mathrm{phs}}$ or ${\hat{s}}_{i}^{\mathrm{phs}}$. Let the matched reference transcript be $T^{*}$. Then, we labeled each junction $e_{j}\in T_{k}$ as a true positive (TP) if $1\left[e_{j}\in T^{*}\right]=1$ and a false-positive (FP) otherwise. Junctions are labeled as false negatives (FN) if they exist in a reference transcript but were not included in any computed transcript.

### 3.2 Data and benchmarking

#### 3.2.1 Simulated data

We simulated RNA-seq data from protein coding genes by randomly sampling from the GENCODE comprehensive gene annotation version V34 (human genome version GRCh38) (Frankish *et al.* 2019) using the Polyester simulator (Frazee *et al.* 2015). We considered 60 randomly sampled genes in each of the following categories of alternative transcript counts ∈{2,3,5,7,10,15,25} (420 genes in total). For each gene, we simulated 50× coverage for eight groups of 100 samples each with different fold changes (1, 1, 1, 1.1, 1.25, 1.5, 3, and 5) to allow for estimation of differential splicing sensitivity (Li *et al.* 2018). We created two additional datasets at 25× and 5× coverage by randomly downsampling sequence reads. The number of reads varied per sample based on a negative binomial distribution for read counts (Frazee *et al.* 2015). The output FASTA files from polyester were aligned to the human genome (GRCh38) using STAR aligner (v. 2.7.3a) with default parameters and GENCODE V34 annotations (Dobin *et al.* 2013). In total, we simulated 1260 genes (420 genes × 3 coverages) yielding over one million synthetic samples.

#### 3.2.2 Experimental data

In addition to simulated, we also validated BSEEJ on bulk RNA-seq data from lymphoblastoid cell lines (GEUVADIS) (Lappalainen *et al.* 2013) and RNA sequencing of monocytes in response to bacterial and viral stimuli (EGAS00001001895) (Rotival *et al.* 2019). GEUVADIS contains 465 individuals that span five populations: Utah residents with northern and western European ancestry, Finnish from Finland, British from England and Scotland, Toscani from Italia, and Yoruba from Ibadan, Nigeria; each population consists of 89−95 samples. The EGA data contains 200 males divided into two 100 individual groups separated by self-reported African or European ancestry (Rotival *et al.* 2019). Up to five samples from peripheral blood mononuclear cells were collected for each individual resulting in 970 total samples. One sample remained untreated, while the four other samples were exposed over 6 h to bacterial lipopolysaccharide, synthetic triacylated lipopeptide (Pam_3_CSK_4_), imidazoquinoline compound (R848), and human seasonal influenza A virus.

### 3.3 Preprocessing and model selection

For each gene, we extracted junction reads based on junctions identified using RegTools (v. 0.5.1). The intervals of genes that overlap were combined. We refined the set of junctions by removing reads that do not map uniquely (e.g. due to paralogous genes), short introns (<50 bp), long introns (>500 000 bp), and FP splice junctions identified by Portcullis (Mapleson *et al.* 2018). While transcript annotations were not used, they could be leveraged to filter out potentially irrelevant or false-positive junctions. Data processing commands can be found in the Supplementary Materials (Supplementary Section Sample Processing, available as supplementary data at *Bioinformatics* online).

We selected the number of SEEJs *K* based on the structure of the graph *G*. The minimum number of SEEJs is equal to the chromatic number of *G* (denoted χ(G)). Since *G* is an interval graph, the chromatic number is equal to the number of vertices in the maximum clique and can be computed in polynomial time (Marathe *et al.* 1992). Then, for each gene, we trained BSEEJ with K=χ(G)+τ, where τ∈{0,2,4,6,8,10,12,14,16} and selected the model with the highest predictive likelihood in held-out validation data. For hyperparameter selection, we implemented a grid search on held-out genes to select hyperparameters where α∈{0.001,0.01,1,5,10}, η∈{0.01,1,5,10}, r∈{1,5,10}, and r=s. Model selection yielded η=0.01 and α=r=s=1 for both simulated and experimental data. We used relative fixed-width stopping rules to assess model convergence (Flegal and Gong 2015), which evaluates the width of the log-likelihood confidence interval relative to a threshold (here, σ=0.001). We used default parameters for competing methods. The number of model variables in the fitted model were similar across each dataset, though the simulated data produced a larger diversity of the number of unique intron excisions and model sizes (Fig. S2, available as supplementary data at *Bioinformatics* online).

**Figure 2.**
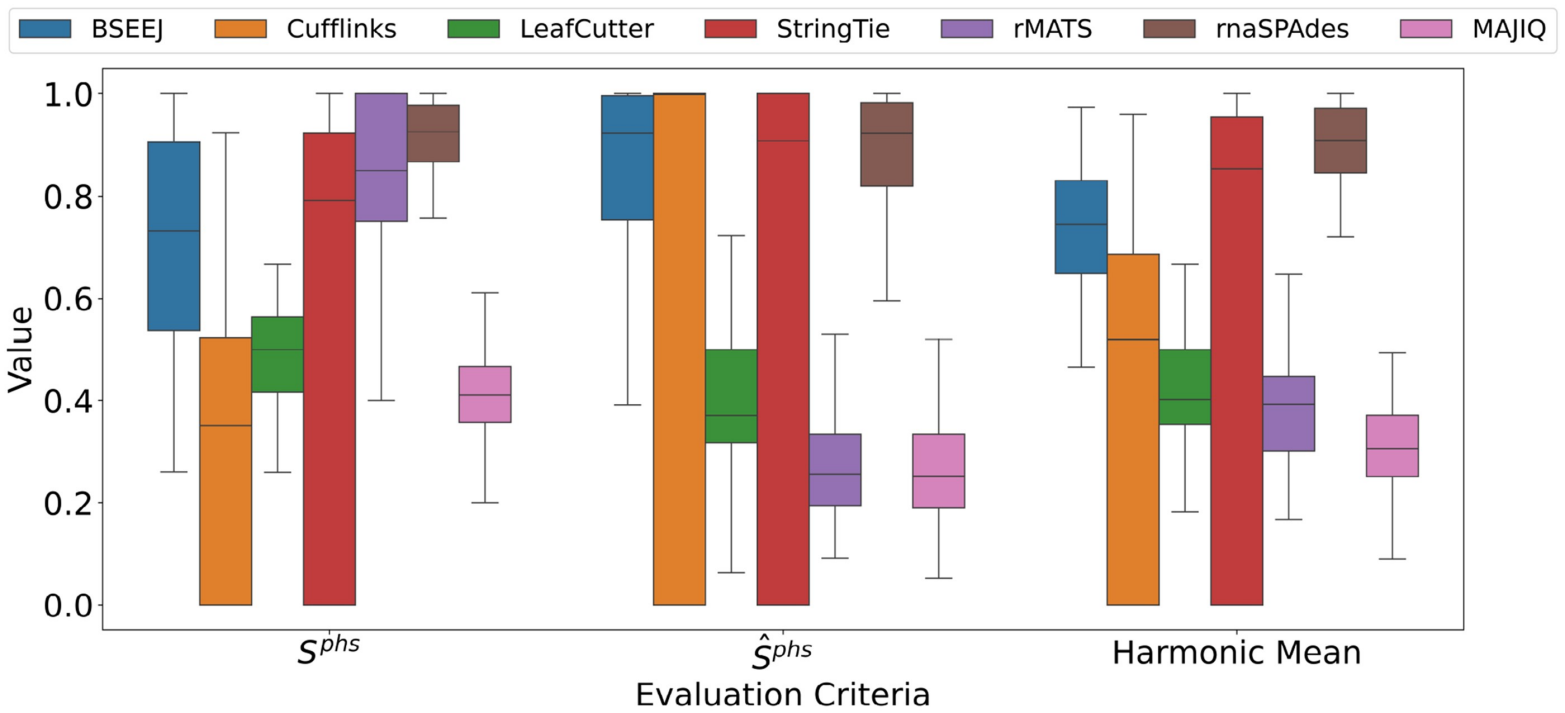
Transcript segment matching to reference. Box plots for $s^{\mathrm{phs}}$, ${\hat{s}}^{\mathrm{phs}}$, and their harmonic mean across the methods in the simulated data. Each box shows the median, interquartile range (IQR), and Tukey whiskers (1.5*IQR from the nearest hinge).

### 3.4 Evaluating reconstruction

We evaluated the transcript reconstruction performance of all methods with respect to partial homogeneity scores (Fig. 2). Due to the computational demands of rnaSPAdes (each gene took ≈3 days), we restricted the following reconstruction evaluations to a subset of 20 randomly selected genes per fold change and transcript count, but include the results on the full data in the Supplementary Materials, available as supplementary data at *Bioinformatics* online.

Splice events produced by LeafCutter, rMATS, and MAJIQ matched with a higher $s^{\mathrm{phs}}$ score compared to their ${\hat{s}}^{\mathrm{phs}}$ scores because the latter normalization is based on the number of junctions in the reference transcript. Since Cufflinks and StringTie both aim to reconstruct full-length transcripts, their ${\hat{s}}^{\mathrm{phs}}$ scores are comparatively higher than splice event methods; however, the Cufflinks $s^{\mathrm{phs}}$ score is considerably lower, indicating that computed transcripts combined junctions that do not exist in the reference. Interestingly, StringTie did not suffer from the same decrease in performance as Cufflinks, though both StringTie and Cufflinks exhibited high variance in their scores. In comparison, BSEEJ demonstrated less variability in $s^{\mathrm{phs}}$ and ${\hat{s}}^{\mathrm{phs}}$ than Cufflinks and StringTie, while maintaining high performance across both scores. This result supports our Bayesian model construction, since BSEEJ appears to focus on reconstructing SEEJs that allow for high probability junction read assignment regardless of SEEJ length. Lastly, rnaSPAdes achieved the highest mean score, suggesting that *de novo* assembly of transcripts might have advantages over read alignment-based methods with respect to segment matching. Results on the full simulated data (without rnaSPAdes) demonstrated similar trends (Fig. S3, available as supplementary data at *Bioinformatics* online).

**Figure 3.**
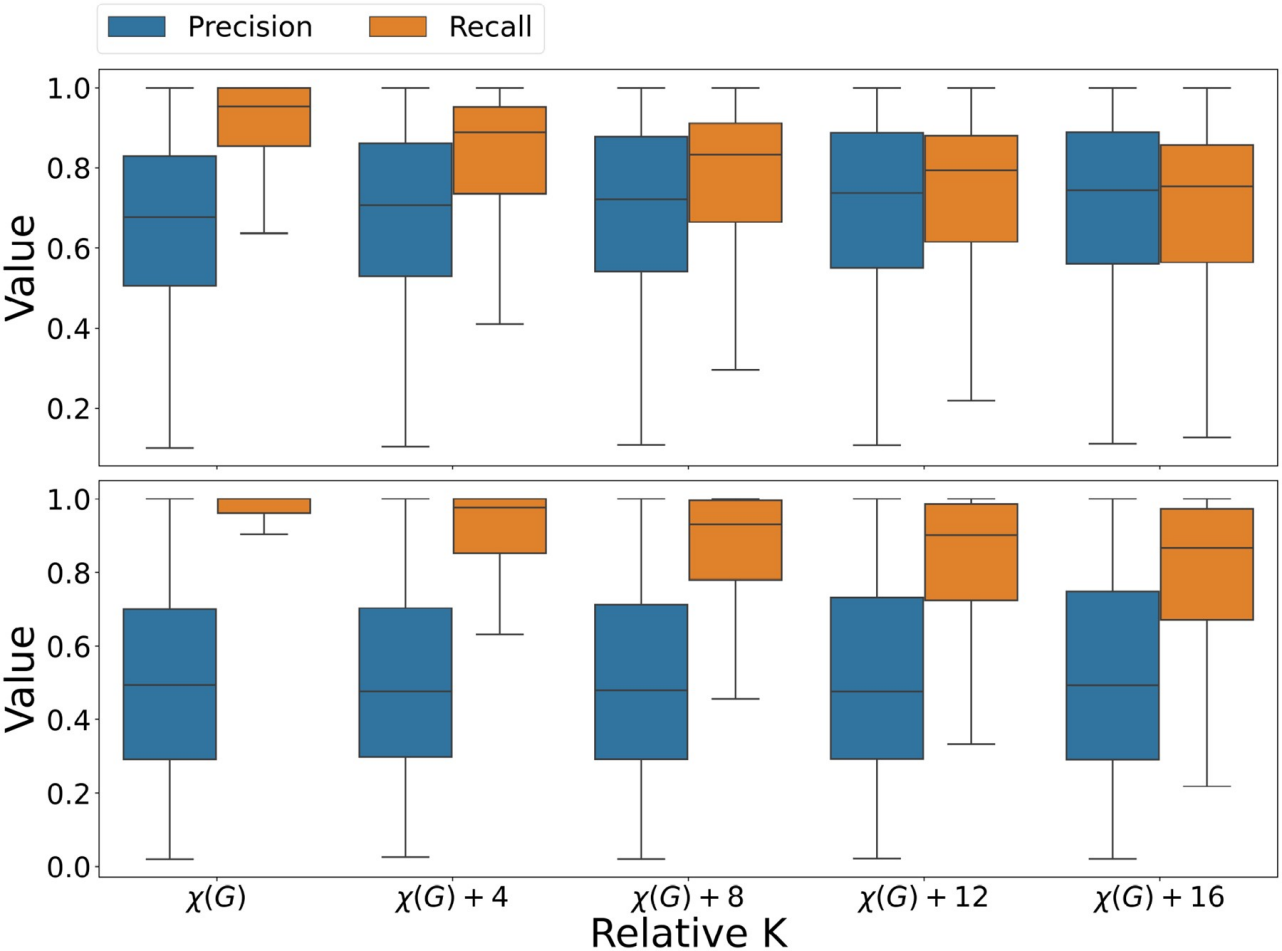
Precision and recall for $s^{\mathrm{phs}}$ (top) and ${\hat{s}}^{\mathrm{phs}}$ (bottom) in models where K=χ(G)+{0,4,8,12,16}. Box show the median, IQR, and Tukey whiskers (1.5*IQR from the nearest hinge).

Next, we evaluated the impact of *K* (the number of SEEJs) on SEEJ reconstruction by varying *K* from χ(G) to χ(G)+16; the empirical distribution of χ(G) across genes was positively skewed, suggesting that alternative transcripts shared different junction conflicts (Fig. S4, available as supplementary data at *Bioinformatics* online). The trend for both $s^{\mathrm{phs}}$ (Fig. 3, top and Fig. S5, top, available as supplementary data at *Bioinformatics* online, top) and ${\hat{s}}^{\mathrm{phs}}$ (Fig. 3, bottom and Fig. S5, bottom, available as supplementary data at *Bioinformatics* online, bottom) are similar: as *K* increases, precision increases initially and then remains flat while recall decreases monotonically. This is likely due to two factors. First, BSEEJ collapses SEEJs with the same junction configuration after convergence. This means that BSEEJ learns a smaller *effective K* when *K* is set to be larger than the number of alternative transcripts. Second, BSEEJ benefits from the flexibility of additional SEEJs initially, but eventually when K≫χ(G), BSEEJ computes SEEJs that are low abundance and noisy.

**Figure 4.**
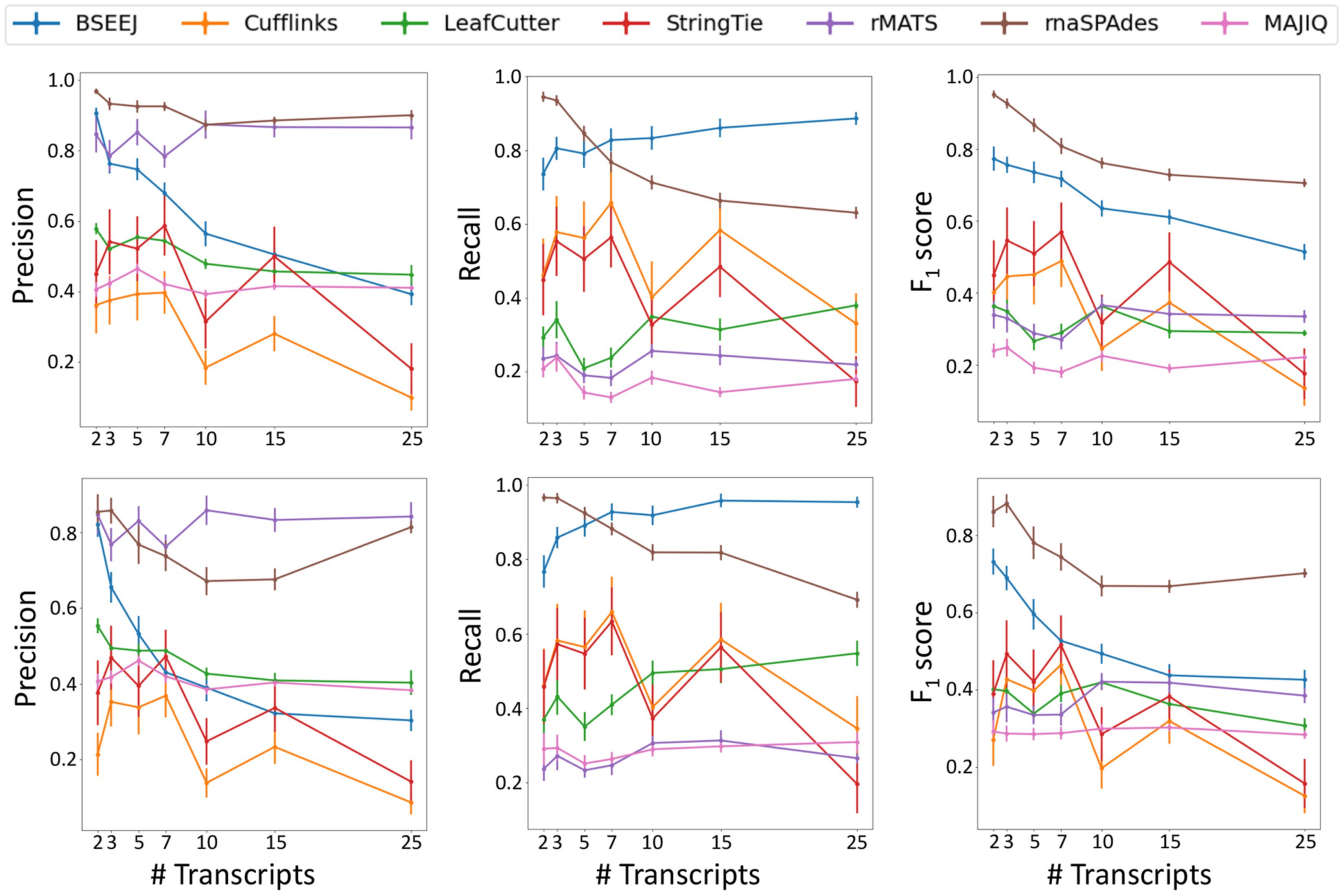
Transcript reconstruction performance on simulated data. Precision, recall, and $F_{1}$ score on simulated data for BSEEJ (blue), Cufflinks (orange), LeafCutter (green), StringTie (red), rMATS (purple), rnaSPAdes (brown), and MAJIQ (pink) based on (top) $S^{\mathrm{phs}}$ and (bottom) ${\hat{S}}^{\mathrm{phs}}$.

**Figure 5.**
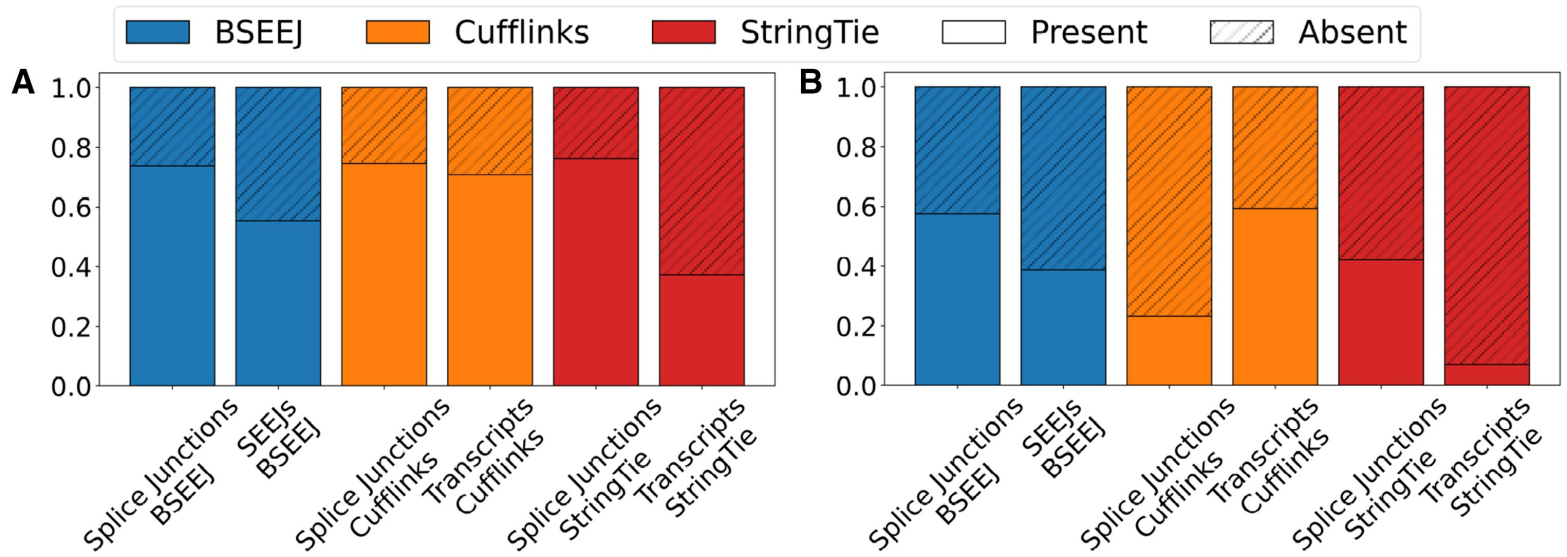
The % of junctions and SEEJs or transcripts that are present or absent in the reference annotations across BSEEJ, Cufflinks, and StringTie for the experimental data. The two plots show the % of present or absent junctions for SEEJs or transcripts in (A) GEUVADIS and (B) EGA.

We next evaluated each method with respect to precision, recall, and $F_{1}$ score by comparing the computed junctions to the reference transcript identified as the maximum $s^{\mathrm{phs}}$ (Fig. 4, top) and ${\hat{s}}^{\mathrm{phs}}$ (Fig. 4, bottom). First, rMATS is highly selective, exhibiting high precision regardless of the number of alternative transcripts. Since rMATS considers a subset of splicing events, the reconstruction task is less difficult; however, for similar reasons, rMATS exhibited a consistently low recall. In contrast, LeafCutter performs local assembly of splicing events into clusters resulting in a comparatively larger recall but smaller precision than rMATS (though the $F_{1}$ score is markedly similar). Cufflinks and StringTie exhibited high variance, but outperformed the local splicing methods in terms of recall since they reconstruct more junctions. The rnaSPAdes assembler achieved the highest overall $F_{1}$ score and precision, at the expense of decreased recall at higher transcript counts. BSEEJ is situated between local splicing and full-length transcript methods and, besides the *de novo* assembler rnaSPAdes, achieved consistently higher precision than the full-length transcript methods and higher recall and $F_{1}$ score with lower standard errors than all non-assembly-based methods. Results on the full simulated dataset, including $F_{1}$, $F_{0.5}$, and $F_{2}$ scores, reflected the same patterns (Figs S6 and S7, available as supplementary data at *Bioinformatics* online).

We also evaluated the precision, recall, and $F_{1}$ score on the full data as a function of transcript complexity (i.e. the number of edges in the excision graph; see Supplementary Section *Combinatorial Model for SEEJ Structure* for details). For genes yielding complex graphs ($|E_{g}|>200$), BSEEJ achieved the highest recall and $F_{1}$ score, while rMATS was the most precise (Fig. S8, available as supplementary data at *Bioinformatics* online). Importantly, this shows that BSEEJ performs well when there is substantial overlap among the transcripts. As a function of the number of excisions, BSEEJ also achieved the highest recall (Fig. S9, available as supplementary data at *Bioinformatics* online). The flexibility of BSEEJ’s admixture model produces high confidence transcript segments rather than fixing the size (e.g. individual splice events or full-length transcripts).

Next, we tested the sensitivity of BSEEJ to model hyperparameters; in particular, we evaluated how the mean posterior SEEJ length (denoted |*SEEJ*| and quantified by the number of junctions) varied as a function of K∈{IS,IS+5,IS+10,IS+15} and r,s∈{0.1,1,10,100}. While the parameter *K* was uncorrelated with |*SEEJ*| (likely due to BSEEJ collapsing posterior SEEJs with the same junctions), we did observe an increase in |*SEEJ*| as the prior mean of Beta(r,s) increased (Fig. S10, available as supplementary data at *Bioinformatics* online). This behavior is consistent with the interpretation of *r* and *s* in the model: *r* and *s* control the prior probability of including a junction in a SEEJ. As the mean of Beta(r,s) increases, larger SEEJs become more likely in the posterior. However, this relationship is not strictly monotonic, as other model parameters, properties of the transcripts, and the stochasticity of model inference interact with the effect of *r* and *s* on |*SEEJ*|. We also observed that training BSEEJ is efficient, with an average per-iteration runtime of 4.037 s. However, the per-iteration runtime increased linearly with *K* (Pearson correlation r=0.565), |*C*| (r=0.721), and the mean number of junction reads in samples (r=0.525; Fig. S11, available as supplementary data at *Bioinformatics* online). Runtime plots on a sample of 10 genes at 5× coverage revealed that StringTie, rnaSPAdes, and MAJIQ were orders of magnitude slower than the competing methods (Fig. S12, available as supplementary data at *Bioinformatics* online).

### 3.5 Differential expression analysis

In the synthetic data, we computed differential expression for all combinations of fold change pairs (28 in total) and focused on BSEEJ, StringTie, rMATS, and LeafCutter, due to the relatively low performance of MAJIQ on our simulated data and the high computational cost for Cufflinks and rnaSPAdes (see Supplementary Section Details on Differential Expression Analysis, available as supplementary data at *Bioinformatics* online). In the synthetic data, BSEEJ achieved the highest sensitivity and accuracy of identifying differential splicing (Table 1), though StringTie achieved the highest specificity (0.996).

**Table 1.** Differential splicing results on simulated data. Bolded values denote the best performance for the corresponding measure.

Method	Accuracy	Sensitivity	Specificity
StringTie	0.253	0.164	0.996
rMATS	0.130	0.0284	0.990
LeafCutter	0.131	0.0292	0.980
BSEEJ	0.303	0.233	0.889

In the experimental data, we identified 3983 and 4278 expressed genes with at least one conflict in *G* in the GEUVADIS and EGA data, respectively. We applied BSEEJ to these data and set K=χ(G)+4 based on simulation results. Using our results on the precision and recall for BSEEJ with varying *K* (Fig. 3), we set K=IS+4. We then applied our Dirichlet-multinomial model to compute differential SEEJ usage. We used the super population (African versus European) and treatment status to group samples in the GEUVADIS and EGA dataset, respectively. After multiple comparisons corrections, we observed 2105 and 1961 genes with significant differential SEEJ usage in the GEUVADIS and EGA data (Benjamini–Hochberg FDR corrected *P* < .05; Fig. S13, available as supplementary data at *Bioinformatics* online) (Benjamini and Hochberg 1995).

We interpreted differential SEEJs by conducting a gene ontology (GO) analysis using genes with differential SEEJ expression as the target list and all genes input into BSEEJ as the background list (Eden *et al.* 2009). In the GEUVADIS data, the top 12 GO terms in the biological process ontology ranked by *P*-value referenced regulation of biomolecular processes (*P* $<2.97\times 10^{-6}$). This is consistent with a growing body of evidence that suggests splicing plays a major role in regulating gene expression (Gehring and Roignant 2021) and metabolism (Kozlovski *et al.* 2017). In the molecular function ontology, alternative splicing plays an integral role in the top 18 GO terms, which reference ATP, DNA, drug, and other molecular binding functions (*P* $<5.69\times 10^{-5}$) (Sciarrillo *et al.* 2020). In the EGA data, both molecular function and biomolecular processes exhibited significant associations with regulation of and binding to kinase proteins (GO:0046330, GO:0043507, GO:0046328, GO:0019901; *P* $<9.7\times 10^{-4}$). Alternative splicing is known to (i) regulate the binding of kinase proteins (Kelemen *et al.* 2013) and (ii) increase kinase protein diversity (Anamika *et al.* 2009). In addition, we computed precision, recall, and $F_{1}$ score by comparing reconstructed SEEJs to GENCODE annotations for the GEUVADIS and EGA datasets (Fig. S14, available as supplementary data at *Bioinformatics* online). Similar to the synthetic results, recall remained higher than precision in each experimental setting.

### 3.6 Novel splice junctions, SEEJs, and transcripts

We quantified the total number and percentages of novel versus known transcript segments in the GEUVADIS and EGA datasets. Since our processing pipeline identifies junctions in a similar manner to the splice event methods, we compared our results to Cufflinks and StringTie. To remove noisy SEEJs, we only considered SEEJs that are expressed in 10 or more samples, where the *k*th SEEJ is considered expressed in sample *i* if there exists 10 or more $z_{ij}=k$. Inferred junction locations were allowed to differ by at most six nucleotide bases from the reference to be considered matching (Li *et al.* 2018). We followed the recommended pipelines for StringTie and Cufflinks and merged per-sample assemblies. A transcript segment was considered novel if it was not a subset of an annotated transcript (and known otherwise). Splice junctions are considered novel if they do not exist in the reference (and known otherwise).

All methods produced fewer novel SEEJs, transcripts, and splice junctions in GEUVADIS compared to the EGA data, which may be due to the relative quality of the lymphoblastoid cell transcriptome compared to monocytes (Fig. S15). BSEEJ and Cufflinks produced larger proportions of known to novel transcript segments in GEUVADIS whereas StringTie generated far more novel transcripts (Fig. 5). This discrepancy was larger in the EGA data, where StringTie produced higher proportions of novel transcripts (<5% were observed in the reference). These results suggest that BSEEJ is more conservative in the identification of novel junctions while simultaneously maintaining the ability to compute novel SEEJs. Finally, to evaluate the performance of BSEEJ as a function of the number of samples, we computed Spearman correlation and Kendall’s τ using $F_{1}$ score and the number of samples per gene (Fig. S16, available as supplementary data at *Bioinformatics* online). We found no substantial association, which is supported by prior work showing that hierarchical Bayesian models are robust to smaller sample sizes (Gelman *et al.* 2013, Aguiar *et al.* 2018, McElreath 2020).

## 4 Discussion and conclusions

In this work, we formalized the SEEJ reconstruction problem, which bridges between the local splicing and full-transcript views of alternative splicing. Then, we developed a hierarchical Bayesian model for discovering SEEJs (BSEEJ) and a GLM to quantify differential SEEJ usage. Both the BSEEJ model and inference algorithm require combinatorial algorithms to solve node cover and independent set problems and to mitigate non-identifiability issues. BSEEJ demonstrated accurate SEEJ reconstruction and differential expression identification compared to six representative splice event detection and transcript reconstruction methods on simulated and experimental datasets.

Our model and problem formulations offer several advantages compared to conventional approaches. First, BSEEJ posits a hierarchical Bayesian model where local SEEJs are drawn from a global SEEJ distribution, which is learned based on all samples simultaneously (not *post hoc*); this enables BSEEJ to focus only on SEEJs with high probability in the posterior of all samples. Second, interpretable model parameters allow for adjusting transcript segment lengths and differential testing of SEEJs. Moreover, similar to LeafCutter, BSEEJ is less affected by the complexities in quantifying relative transcript or exon usage in complex splicing scenarios, focusing instead on excised introns. Lastly, SEEJs provide a succinct, intermediate-level representation of transcript complexity; they can summarize a set of closely related transcripts, reducing the redundancy of enumerating all possible full-length transcripts (Vaquero-Garcia *et al.* 2016, Li *et al.* 2018).

There are several directions for future work, both in terms of SEEJ modeling and addressing limitations of BSEEJ. First, using nonparametric priors on both the individual specific ($\theta_{i}$) and global ($\beta_{k}$) SEEJ distributions would enable (i) model training without costly hyperparameter selection of *K* and (ii) model adaptation to new samples, e.g. from a different tissue or disease condition (Teh *et al.* 2006). Second, modeling of sparse count-based single cell RNA-seq data could be accommodated with different data likelihoods. Lastly, SEEJ-QTLs could test how genetic variants affect SEEJ usage and are a natural analog to splicing QTLs (GTEx Consortium 2015) and transcript ratio QTLs (Lappalainen *et al.* 2013).

## Supplementary Material

btaf646_Supplementary_Data

## Data Availability

Data from the GEUVADIS project are available through ArrayExpress database (www.ebi.ac.uk/arrayexpress) under accession number E-GEUV-6. Data from the EGA project are available through European Genome-Phenome Archive (ega-archive.org) under Study ID: EGAS00001001895. The source code for BSEEJ is freely available at https://github.com/bayesomicslab/BSEEJ.
