# Supplementary material for: Bayesian reconstruction and differential testing of excised introns
Source: Bioinformatics. 2025 Dec 1;41(12):btaf646. doi: 10.1093/bioinformatics/btaf646 (PMC12721381; doi:10.1093/bioinformatics/btaf646)
Supplement: btaf646_Supplementary_Data [file btaf646_supplementary_data.pdf]

Supplementary Materials

Related Work

Methods for alternative splicing (AS) characterization can be loosely grouped into three categories based on their assumed input annotations: no reference annotations (*de novo* assembly), gene transcripts and their exon composition (transcript annotation-based), and gene starting and ending positions only (transcript annotation-free). *De novo* transcriptome assembly methods, like Trinity [Haas et al., 2013] and ABySS [Birol et al., 2009], compute transcripts from unaligned sequence reads, typically without the benefit of reference annotations. When a reference genome sequence is well characterized, transcript annotation-based and annotation-free methods have been shown to produce more accurate transcripts and quantifications [Marchant et al., 2016]; since our focus is on species with well-categorized genome sequences, we restrict our attention to methods that assume sequences reads can be mapped to a genome reference.

Transcript annotation-based and annotation-free isoform reconstruction methods begin by aligning RNA-seq reads to a reference genome using a splice-aware aligner [Dobin et al., 2013, Langmead and Salzberg, 2012]. The overwhelming majority of these methods reconstruct full-length transcripts as ordered sets of exons, focusing on the RNA that is retained. The Bayesian isoform discovery and individual specific quantification (BIISQ) method models transcript reconstruction with a nonparametric Bayesian hierarchical model, where samples are mixtures of transcripts sampled from a population transcript distribution [Aguiar et al., 2018]. While BIISQ was shown to have high accuracy on low abundance isoforms, it requires both the genes and the composite exon coordinates, and is unable to construct isoform transcripts that deviate from this reference annotation. Cufflinks and StringTie construct full-length transcripts and can operate both with or without transcript annotations. Cufflinks reconstructs transcripts as minimum paths in an associated graph, where the aligned reads are vertices, and edges denote the compatibility of isoforms [Trapnell et al., 2010]. StringTie models transcript reconstruction using maximum network flow on a splice graph, where paths and read coverage inform isoform composition and quantification respectively [Pertea et al., 2015]. Both are well-established state-of-the-art methods, but consider samples individually during the initial reconstruction. Additionally, all aforementioned methods are restricted to constructing full-length isoforms, a problem that is made challenging by exon boundaries that are difficult to identify and variability in read depths across transcripts. SEEJ reconstruction naturally bridges the gap between full-length transcript reconstruction methods and single-junction analyses. In genomic loci with complex and highly localized alternative splicing such as the Human Leukocyte Antigen (HLA) region, assembling complete transcripts is extremely challenging due to extensive polymorphisms and closely related isoforms [Lappalainen et al., 2013, Robinson et al., 2015], while single-junction methods may overlook crucial contextual relationships between adjacent splicing events [Li et al., 2018, Shen et al., 2014]. By explicitly modeling sequences of exon-exon junctions, our method effectively addresses these intermediate-scale splicing scenarios.

A more recent class of isoform reconstruction and quantification methods focus on characterizing local splicing events. The local splicing and hierarchical model rMATS detects differential usage of exons through the comparison of exon-inclusions in junction reads among five different alternative splicing events [Shen et al., 2014]. MAJIQ defines local splicing variations (LSV) as regions where multiple distinct splicing events are observed; the method then detects and quantifies differential LSVs [Vaquero-Garcia et al., 2016, 2023]. Interestingly, LeafCutter focuses on introns rather than the constituent exons of a transcript to identify local splicing events. First, LeafCutter computes local splicing events from RNA-Seq data then constructs a graph $G_L = (V_L, E_L)$ where vertices, V_L, are junctions and edges, E_L, connect junctions that share a donor or acceptor splice site [Li et al., 2018]. Subsequently, differential splicing of introns in the connected components of G_L is computed using a Dirichlet-multinomial generalized linear model on read counts. LeafCutter does not suffer the same disadvantages of methods that use exonic sequences or attempt to reconstruct full-length transcripts, though at the expense of the inability to identify certain splicing events like alternative transcription start sites. These methods also may fail to capture interactions between splicing events on the same transcript and may conflate transcripts that share splice events. For example, if two transcripts share an intron, the read counts on the shared junction will be summed conflating the two transcripts and potentially masking differential expression across two populations (Figure 1C).

Our method, BSEEJ, is situated in between full-length transcript and local splicing methods (Fig. 1). BSEEJ benefits from the transcript annotation-free nature of junctions, while also being able to support both local splicing events, full-length transcripts, and variable lengths in between. BSEEJ also shares similarities with BIISQ in that it considers all samples jointly and is defined as a formal probabilistic model, enabling the quantification of uncertainty and direct interpretation of fitted model parameters that are used to both explore the results and develop a method for differential testing.

Additional Model Details

Notations

Variables and indices, parameters and hyper-parameters and sets are as following:

- V is the set of unique junctions, indexed by v and its size of denoted by $|V|$.
- N is the number of samples and are indexed by i.
- J_i is the number of junctions in ith sample. The junctions of a sample are indexed by j. But the length of all the samples are not necessarily the same. Furthermore, some samples might not have some of the junctions from the set of unique junctions V.
- K is the number of sequences of exon-exon junctions (SEEJs). For the jth junction in the ith sample ($i \in \{1, \ldots, N\}$ and $j \in \{1, \ldots, J_i\}$), we assign a SEEJ $k \in \{1, \ldots, K\}$.
- Graph $G = (V_g, E_g)$, where V_g is the set of junctions and there is an edge between two junctions *iff* their intersection is non-empty.
- Ω is the set of all the independent sets in G.
- $\mathcal{N}_v = \{u | \{u, v\} \in E_g\}$ is the set of all the neighbors of node v in the interval graph G.

- ϕ_k^{it} is the selected configuration as SEEJ k in the iteration it and follows a multinomial distribution ($\sim$ Multinomial$(\phi_{k1}, \phi_{k2}, \ldots, \phi_{kt}, \ldots, \phi_{kT})$).
- C is the set of all Bernoulli random variables required for encoding all conflicts in $G(V_g,E_g)$, and $|C|$ is equal to minimum node cover in G.
- Hyper-parameter $\boldsymbol{\alpha} = (\alpha_1, \ldots, \alpha_K)$ is a K-dimensional vector and prior for θ variable.
- For the ith sample, variable $\theta_i \sim \text{Dirichlet}_K(\boldsymbol{\alpha})$ is a K-dimensional Dirichlet distribution and represents the proportions of the SEEJs in sample i. So $\boldsymbol{\theta}$ is a $N \times K$ matrix such that each row shows the distribution of SEEJs for a sample and θ_{ik} shows the proportion of SEEJ k in sample i ($\boldsymbol{\theta} \in \mathbb{R}^{N \times K}$).
- Variable z_{ij} is the SEEJ assignment for jth junction in ith sample. It can take a natural value between 1 and K and follows a multinomial distribution ($\boldsymbol{Z} \in \{1, \ldots, K\}^{N \times J}$ and $z_{ij} \sim \text{Multinomial}(\theta_i)$).
- Hyper-parameters r and s are priors for π beta distribution.
- Variable $\pi_k \sim \text{Beta}(r,s), \forall k = \{1, \ldots, K\}$, so $\boldsymbol{\pi}$ is a K-dimensional vector and prior for Bernoulli variable $\boldsymbol{b}$.
- Hyper-parameter $\boldsymbol{\eta} = (\eta_1, \ldots, \eta_{|V|})$ is a $|V|$-dimensional vector and prior for β variable.
- For SEEJ k, $\beta_k \sim \text{Dirichlet}_{|V|}(\boldsymbol{\eta} \odot \boldsymbol{b_k})$ is a $|V|$-dimensional Dirichlet which represents the distribution of the SEEJ k over the junctions. $|V|$-dimensional vector $\boldsymbol{b_k} = (b_{k1}, \ldots, b_{k|V|})$ (also written as $\boldsymbol{b_{k.}}$) is the kth row of the $\boldsymbol{b}$ matrix and collects the Bernoulli variables for all the unique junctions. The *dot* in $\boldsymbol{b_{k.}}$ means all the unique junctions in row k. Notation $\odot$ is element-wise multiplication. Matrix $\boldsymbol{\beta}$ is $K \times |V|$ and the element in kth row and vth columns shows the proportion of junctions v in SEEJ k, so matrix $\boldsymbol{\beta} \in \mathbb{R}^{K \times |V|}$. Note that the Bernoulli variables can turn off/on certain dimensions of β_k variables.
- In the ith sample, the jth junction is w_{ij} and is observed and follows a multinomial distribution ($w_{ij} \sim \text{Multinomial}(\beta_{z_{ij}})$), so matrix $\boldsymbol{W}$ is a $N \times J$ ($W \in \{1, \ldots, |V|\}^{N \times J}$) and w_{ij} is the element in ith row and jth column of $\boldsymbol{W}$ and is jth junction in ith sample and is observed. Note that in $\boldsymbol{W}$, row i correspond to sample i, but not all the rows have the same number of columns due to the differences between the number of junctions in different samples, *i.e.* row i has exactly J_i columns (elements) which correspond to the junctions in the sample i. We call $\boldsymbol{W}$ here as a matrix for the ease of notation, but it is actually a list of list. The same explanation applies to matrix $\boldsymbol{Z}$ too.
- $\oplus$ is exclusive OR.
- $\odot$ is element-wise vector multiplication.

Graphical and Generative Models

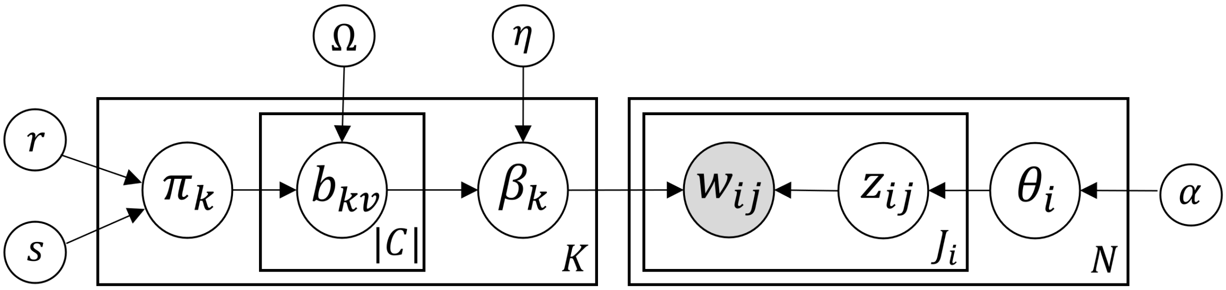

Fig. S1. Graphical model for BSEEJ. The variables $\boldsymbol{\pi}$, $\boldsymbol{b}$, and $\boldsymbol{\beta}$ control the global sequence of exon-exon junctions (SEEJ) structure, while $\boldsymbol{w}$, $\boldsymbol{z}$, and $\boldsymbol{\theta}$ control the sample-specific distribution of SEEJs.

The BSEEJ generative model is given by

$$
\begin{aligned}
\theta_i &\sim \text{Dirichlet}_K(\boldsymbol{\alpha}), && \forall i \in \{1, \ldots, N\} \\
z_{ij} &\sim \text{Multinomial}_K(\theta_i), && \forall i \in \{1, \ldots, N\}, \forall j \in \{1, \ldots J_i\} \\
w_{ij} &\sim \text{Multinomial}(\beta_{z_{ij}}), && \forall i \in \{1, \ldots, N\}, \forall j \in \{1, \ldots J_i\} \\
\beta_k &\sim \text{Dirichlet}_{|V|}(\boldsymbol{\eta} \odot \boldsymbol{b_k}), && \forall k \in \{1, \ldots, K\} \\
b_{kv} &\sim \text{Bernoulli}(\pi_k), && \forall k \in \{1, \ldots, K\}, \forall v \in C \\
\pi_k &\sim \text{Beta}(r,s), && \forall k \in \{1, \ldots, K\}
\end{aligned}
$$

In the subsequent derivations, the beta function B(.) is given by

$$B(\boldsymbol{\alpha}) = \frac{\prod_{i=k}^{K} \Gamma(\alpha_i)}{\Gamma(\sum_{i=1}^{K} \alpha_i)}$$

where $\Gamma()$ is the Gamma function, $\boldsymbol{\alpha}$ is the vector of Dirichlet concentration parameters, and $K \geq 2$ is the number of SEEJs. $\boldsymbol{C}$ is a $N \times K$ matrix, in which c_{ik} is the number of junctions in the ith sample that have been assigned to SEEJ k. $\boldsymbol{\lambda}$ is a $K \times |V|$ matrix, in which λ_{kv} is the number of times junction v has been assigned to SEEJ k.

Inference - Gibbs Sampling

We fist compute the complete conditionals of all the variables in the model.

Complete Conditional of $\boldsymbol{\theta_i}$

$$\begin{aligned} p(\theta_i|\boldsymbol{\alpha}, z_{i,j=1:J_i}) &\propto p(z_{i,j=1:J_i}, \boldsymbol{\alpha}, \theta_i) \\ &= \prod_{j=1}^{J_i} p(z_{ij}|\theta_i)p(\theta_i|\boldsymbol{\alpha}) \end{aligned} \tag{1}$$

The first term in Eq. 1 is a multinomial and the second term is a K-dimensional Dirichlet. So for updating θ_i we have:

$$p(\theta_i|\boldsymbol{\alpha}, z_{i,j=1:J_i}) \propto \frac{B(\boldsymbol{\alpha} + \boldsymbol{c_{i.}})}{B(\boldsymbol{\alpha})} \times \text{Dirichlet}_K(\boldsymbol{\alpha} + \boldsymbol{c_{i.}})$$

where $\boldsymbol{c_{i.}}$ is a K-dimensional vector containing the proportion of SEEJs in sample i.

$$\boldsymbol{c_{i.}} = [c_{i,k=1}, c_{i,k=2}, \ldots, c_{i,k=K}]$$

Complete Conditional of $\boldsymbol{Z}$

Lets consider complete conditional of one z_{ij} variable (SEEJ assignment of jth junction in i^{th} sample):

$$\begin{aligned} p(z_{ij}|\theta_i, w_{ij}, \boldsymbol{\beta_{1:K}}) &\propto p(z_{ij}, w_{ij}, \theta_i, \boldsymbol{\beta_{1:K}}) \\ &\propto p(w_{ij}|z_{ij}, \boldsymbol{\beta_{1:K}})p(z_{ij}|\theta_i) \end{aligned}$$

The notation $\boldsymbol{\beta_{1:K}}$ means that variable z_{ij} is dependent on β_1 to β_K. Since $z_{ij} \in \{1, 2, \ldots, K\}$ (that is, z_{ij} is a discrete random variable), the complete conditional for an assignment $z_{ij} = k$ would be

$$p(z_{ij} = k|\theta_i, w_{ij}, \boldsymbol{\beta_{1:K}}) = \frac{p(z_{ij} = k|\theta_i)p(w_{ij}|z_{ij} = k, \boldsymbol{\beta_{1:K}})}{\sum_{k=1}^{K} p(z_{ij} = k|\theta_i)p(w_{ij}|z_{ij} = k, \boldsymbol{\beta_{1:K}})}$$

And in sample i, for SEEJ k:

$$\begin{aligned} p(z_{ij} = k|\theta_i, w_{ij}, \boldsymbol{\beta_{1:K}}) &\propto p(z_{ij} = k|\theta_i)p(w_{ij}|z_{ij} = k, \boldsymbol{\beta_{1:K}}) \\ &\propto \theta^{c_{ik}}\theta^{\alpha_i - 1} \times \prod_{v=1}^{|V|} \beta_{kv}^{\lambda_{kv}} \beta^{\eta_{kv} b_{kv} - 1} \end{aligned}$$

The probability of assigning the (unique) junction v (in any position j in sample i) to SEEJ k:

$$p(z_{ij} = k|\theta_i, w_{ij} = v, \boldsymbol{\beta_{1:K}}) = \frac{\theta_{ik}\beta_{kv}}{\sum_{k=1}^{K} \theta_{ik}\beta_{kv}}$$

Complete Conditional of $\boldsymbol{\beta}$

For SEEJ k, β_k is a $|V|$-dimensional Dirichlet distribution over the unique junctions. We used Bernoulli variables b that restrict the number of junctions in one SEEJ (the size of β_k variables) by defining $\beta_k \sim \text{Dirichlet}_{|V|}(\eta_1 b_{k1}, \ldots, \eta_{|V|} b_{k|V|})$. Thus, β_k is a degenerate Dirichlet distribution.

$$\begin{aligned} p(\beta_k|\boldsymbol{W}, \boldsymbol{Z}, \boldsymbol{b_{k.}}) &\propto p(\beta_k, w_{..}, z_{..}, \boldsymbol{b_{k.}}) \\ &= p(w_{..}|z_{..}, \beta_k)p(\beta_k|\boldsymbol{b_{k.}}, \boldsymbol{\eta}) \end{aligned}$$

For SEEJ k:

$$\begin{aligned} p(\beta_k|\boldsymbol{W}, \boldsymbol{Z}, \boldsymbol{b_{k.}}) &\propto \prod_{v=1}^{V} \beta_{kv}^{\lambda_{kv}} \times \frac{\Gamma(\sum_{v=1}^{V} \eta_v b_{kv})}{\prod_{v=1}^{V} \Gamma(\eta_v b_{kv})} \times \prod_{v=1}^{V} \beta_{kv}^{\eta_v b_{kv} - 1} \\ &\propto \prod_{v=1}^{V} \beta_{kv}^{\lambda_{kv}} \times \beta_{kv}^{\eta_v b_{kv} - 1} \\ &\propto Dir_{|V|}(\boldsymbol{\lambda_{k.}} + \boldsymbol{\eta} \odot \boldsymbol{b_{k.}}) \end{aligned}$$

In which vector $\boldsymbol{\lambda_{k.}}$ is the count of junctions that have been assigned to SEEJ k:

$$\boldsymbol{\lambda_{k.}} = [\lambda_{k,v=1}, \ldots, \lambda_{k,v=|V|}]$$

Complete Conditional of $\boldsymbol{b}$

Let the space Ω span over the set of all independent sets in the junction interval graph G (see §*The BSEEJ Model*). Here, we consider computing the Gibbs updates for independent sets $\{\Phi_1,\dots,\Phi_T\} \in \Omega$. For ease of exposition, we consider a b_{kv} given a configuration $\hat{\Phi} \in \Omega$. For a junction v, we compute the probability of occurrence of v in SEEJ k. This probability is obtained by the complete conditional of b_{kv}. We note that, for computing b_{kv}, we need to consider only the relevant dimensions of the Dirichlet. For example, in a SEEJ k, for the calculation of complete conditional for $b_{kv} = 1$, such dimensions include all the junctions that are not in the neighborhood of v ($\{v' | v' \notin \mathcal{N}_v\}$), where $\mathcal{N}_v$ is the set of all the neighbors of v and does not include v itself (open neighborhood). We denote $\boldsymbol{b}^{(-kv)}$ as the vector $\boldsymbol{b}$ with b_{kv} removed and suppress hyperparameters for readability when appropriate.

$$
\begin{aligned}
p(b_{kv} = 1 | \boldsymbol{\beta}, \boldsymbol{\pi}, \boldsymbol{b}^{(-kv)}, \boldsymbol{W}, \boldsymbol{Z}, \boldsymbol{\theta}) &\propto p(\boldsymbol{\beta}, \boldsymbol{\pi}, \boldsymbol{b}, \boldsymbol{W}, \boldsymbol{Z}, \boldsymbol{\theta}) \\
&\propto p(b_{kv} = 1 | \beta_k, \pi_k, \boldsymbol{b_{k\cdot}}) \\
&= p(b_{kv} = 1 | \pi_k) p(\pi_k | r, s) p(\beta_k | \boldsymbol{b_{k\cdot}}, \boldsymbol{\eta}) \\
&= p(b_{kv} = 1 | \pi_k) \frac{\Gamma(r+s)}{\Gamma(r)\Gamma(s)} \pi_k^{r-1} (1-\pi_k)^{s-1} \\
&\quad \frac{\Gamma(\sum_{i \in \hat{\Phi} \cup \{b_{kv}\}} \eta_i b_{ki})}{\prod_{i \in \hat{\Phi} \cup \{b_{kv}\}} \Gamma(\eta_i b_{ki})} \prod_{i \in \hat{\Phi} \cup \{b_{kv}\}} \beta_{ki}^{\eta_i b_{ki} - 1} \\
&\propto p(b_{kv} = 1 | \pi_k) \pi_k^{r-1} (1-\pi_k)^{s-1} \\
&\quad \frac{\Gamma(\sum_{i \in \hat{\Phi} \cup \{b_{kv}\}} \eta_i b_{ki})}{\prod_{i \in \hat{\Phi} \cup \{b_{kv}\}} \Gamma(\eta_i b_{ki})} \prod_{i \in \hat{\Phi} \cup \{b_{kv}\}} \beta_{ki}^{\eta_i b_{ki} - 1} \\
&\propto \pi_k \pi_k^{r-1} (1-\pi_k)^{s-1} \\
&\quad \frac{\Gamma(\sum_{i \in \hat{\Phi} \cup \{b_{kv}\}} \eta_i b_{ki})}{\prod_{i \in \hat{\Phi} \cup \{b_{kv}\}} \Gamma(\eta_i b_{ki})} \prod_{i \in \hat{\Phi} \cup \{b_{kv}\}} \beta_{ki}^{\eta_i b_{ki} - 1} \\
&\propto \pi_k^{r} (1-\pi_k)^{s-1} \frac{\Gamma(\sum_{i \in \hat{\Phi} \cup \{b_{kv}\}} \eta_i b_{ki})}{\prod_{i \in \hat{\Phi} \cup \{b_{kv}\}} \Gamma(\eta_i b_{ki})} \prod_{i \in \hat{\Phi} \cup \{b_{kv}\}} \beta_{ki}^{\eta_i b_{ki} - 1}
\end{aligned}
$$

which is the product of a Beta$(r+1,s)$ and a degenerate Dirichlet. In SEEJ k, for computing complete conditional for $b_{kv} = 0$, we need to include the other junctions except v, so:

$$
\begin{aligned}
p(b_{kv} = 0 | \boldsymbol{\beta}, \boldsymbol{\pi}, \boldsymbol{b}^{(-kv)}, \boldsymbol{W}, \boldsymbol{Z}, \boldsymbol{\theta}) &\propto p(\boldsymbol{\beta}, \boldsymbol{\pi}, \boldsymbol{b}, \boldsymbol{W}, \boldsymbol{Z}, \boldsymbol{\theta}) \\
\propto p(b_{kv} = 0 | \beta_k, \pi_k, \boldsymbol{b_{k\cdot}}) &\propto p(b_{kv} = 0 | \pi_k) p(\pi_k | r, s) p(\beta_k | b_{k\cdot}, \eta) \\
= p(b_{kv} = 0 | \pi_k) \frac{\Gamma(r+s)}{\Gamma(r)\Gamma(s)} \pi_k^{r-1} (1-\pi_k)^{s-1} & \frac{\Gamma(\sum_{i \in \hat{\Phi} \setminus \{b_{kv}\}} \eta_i b_{ki})}{\prod_{i \in \hat{\Phi} \setminus \{b_{kv}\}} \Gamma(\eta_i b_{ki})} \prod_{i \in \hat{\Phi} \setminus \{b_{kv}\}} \beta_{ki}^{\eta_i b_{ki} - 1} \\
\propto p(b_{kv} = 0 | \pi_k) \pi_k^{r-1} (1-\pi_k)^{s-1} & \frac{\Gamma(\sum_{i \in \hat{\Phi} \setminus \{b_{kv}\}} \eta_i b_{ki})}{\prod_{i \in \hat{\Phi} \setminus \{b_{kv}\}} \Gamma(\eta_i b_{ki})} \prod_{i \in \hat{\Phi} \setminus \{b_{kv}\}} \beta_{ki}^{\eta_i b_{ki} - 1} \\
\propto (1-\pi_k) \pi_k^{r-1} (1-\pi_k)^{s-1} & \frac{\Gamma(\sum_{i \in \hat{\Phi} \setminus \{b_{kv}\}} \eta_i b_{ki})}{\prod_{i \in \hat{\Phi} \setminus \{b_{kv}\}} \Gamma(\eta_i b_{ki})} \prod_{i \in \hat{\Phi} \setminus \{b_{kv}\}} \beta_{ki}^{\eta_i b_{ki} - 1} \\
\propto \pi_k^{r-1} (1-\pi_k)^{s} & \frac{\Gamma(\sum_{i \in \hat{\Phi} \setminus \{b_{kv}\}} \eta_i b_{ki})}{\prod_{i \in \hat{\Phi} \setminus \{b_{kv}\}} \Gamma(\eta_i b_{ki})} \prod_{i \in \hat{\Phi} \setminus \{b_{kv}\}} \beta_{ki}^{\eta_i b_{ki} - 1}
\end{aligned}
$$

which is the product of a Beta$(r,s+1)$ and a degenerate Dirichlet. In general, we have T independent sets $\{\Phi_1,\dots,\Phi_T\}$ and the update is computed by sampling

$$
\text{Categorical}\left(\frac{p(\Phi_1)}{\sum p(\Phi_{i=1}^{T})}, \dots, \frac{p(\Phi_T)}{\sum p(\Phi_{i=1}^{T})} \right) \tag{2}
$$

We develop algorithm S2 for computing $\{\Phi_1,\dots,\Phi_T\}$ (§S3.8). In the Gibbs updates, first, we compute $p(b_{kv} = 1|\cdot)$ and $p(b_{kv} = 0|\cdot)$. Then, we know that every node that is a neighbor to b_{kv} is currently not selected (evaluates to 0). We compute $p(b_{ki} = 1, b_{kv} = 0)$ for each $i \in \mathcal{N}_v$ if setting $b_{ki} = 1$ yields a valid configuration. Finally, we update SEEJ structure by moving from one independent set to another that is *close* according to Eq. 2.

In the interval graph of junctions $G = (V_g, E_g)$, let Ω be the set of all the independent sets and $\phi_{kt} \subseteq \Omega$ be the tth locally generated valid configuration for SEEJ k. We define the neighbor of ϕ_{kt} as $\mathcal{N}(\phi_{kt})$, i.e. the set of the nodes that intersect with some of the nodes in ϕ_{kt} (or $\mathcal{N}(\phi_{kt}) = \{u | \{u,v\} \in E_g$ for some $v \in \phi_{kt}\}$). Then $p(\phi_{kt})$ is computed as the following:

$$p(\phi_{kt}|\boldsymbol{\beta},\boldsymbol{\pi},\boldsymbol{b},\boldsymbol{W},\boldsymbol{Z},\boldsymbol{\theta}) \propto \pi_k^{r+|\phi_{kt}|-1}(1-\pi_k)^{s+|\mathcal{N}(\phi_{kt})|-1} \frac{\Gamma(\sum_{i\in V\setminus\mathcal{N}(\phi_{kt})}\eta_i b_{ki})}{\prod_{i\in V\setminus\mathcal{N}(\phi_{kt})}\Gamma(\eta_i b_{ki})} \prod_{i\in V\setminus\mathcal{N}(\phi_{kt})} \beta_{ki}^{\eta_i b_{ki}-1}$$

Complete Conditional of π_k

We define m_k equal to the number of junctions that are selected in SEEJ k, *i.e.* the junctions whose corresponding Bernoulli variable is 1 in the current Gibbs iteration:

$$m_k = \sum_{v\in V} \mathbf{1}[b_{kv}=1], \qquad \forall k \in K$$

Then, the complete conditional for π_k can be expressed as

$$\begin{aligned}
p(\pi_k|\boldsymbol{b_{k\cdot}},r,s) &\propto p(\pi_k,\boldsymbol{b_{k\cdot}},r,s) \\
&= p(\pi_k|r,s)p(b_{k\cdot}|\pi_k) \\
&= \frac{\Gamma(r+s)}{\Gamma(r)\Gamma(s)} \times \pi_k^{r-1}(1-\pi_k)^{s-1} \times \pi_k^{m_k}(1-\pi_k)^{|V|-m_k} \\
&\propto \pi_k^{r+m_k-1}(1-\pi_k)^{s+|V|-m_k-1} \\
&= \frac{\Gamma(r+m_k)\Gamma(s+|V|-m_k)}{\Gamma(r+s+|V|)} \times \mathrm{Beta}(r+m_k, s+|V|-m_k) \\
&\propto \mathrm{Beta}(r+m_k, s+|V|-m_k)
\end{aligned}$$

Likelihood

The likelihood is given by

$$\begin{aligned}
p(\boldsymbol{W}|\boldsymbol{\beta},\boldsymbol{Z}) &\propto p(\boldsymbol{W},\boldsymbol{\beta},\boldsymbol{Z}) \\
&\propto p(\boldsymbol{W}|\boldsymbol{\beta},\boldsymbol{Z})p(\boldsymbol{Z}|\boldsymbol{\theta}) \\
&\propto \prod_{i=1}^{N}\prod_{k=1}^{K}\prod_{v=1}^{|V|} \beta_{kv}^{\xi_{kv}^{(i)}}
\end{aligned}$$

where $\xi_{kv}^{(i)}$ is the number of times junction v is assigned to SEEJ k in the sample i.

Gibbs Sampling Algorithm

We sample variables using their complete conditionals as follows:

$$\begin{aligned}
&p(z_{ij}|\theta_i, w_{ij}, \boldsymbol{\beta_{1:K}}) \propto p(w_{ij}|z_{ij},\boldsymbol{\beta_{1:K}})p(z_{ij}|\theta_i) \\
&p(\theta_i|\boldsymbol{\alpha}, z_{i,j=1:J_i}) \propto \prod_{j=1}^{J_i} p(z_{ij}|\theta_i)p(\theta_i|\boldsymbol{\alpha}) \\
&p(\beta_k|\boldsymbol{W},\boldsymbol{Z},\boldsymbol{b_{k\cdot}}) \propto p(w_{\cdot\cdot}|z_{\cdot\cdot},\beta_k)p(\beta_k|\boldsymbol{b_{k\cdot}},\boldsymbol{\eta}) \\
&p(b_{kv}=1|\boldsymbol{\beta},\boldsymbol{\pi},\boldsymbol{b^{(-kv)}},\boldsymbol{W},\boldsymbol{Z},\boldsymbol{\theta}) \propto p(b_{kv}=1|\beta_k,\pi_k,\boldsymbol{b_{k\cdot}}) \\
&p(\pi_k|\boldsymbol{b_{k\cdot}},r,s) \propto p(\pi_k|r,s)p(\boldsymbol{b_{k\cdot}}|\pi_k)
\end{aligned}$$

Local Search Algorithm

We are given the proportion of junctions in SEEJ k β_k, the number of sampled local independent sets T, the set of nodes in SEEJ k (current configuration) S, the interval graph of the junctions G, and the size of maximum clique in the complement graph of G $\omega(\bar{G})$. Then, Algorithm S2 outputs set Φ, which includes T local independent sets. Since G is an interval graph, independent sets can be computed efficiently [Andrade et al., 2012]. The algorithm first decides whether to add or remove elements from the current configuration by sampling from a Bernoulli which is proportional to the size of current configuration. Then, junctions are selectively added or removed with probability proportional to β_k, which is justified through the conditional dependencies defined in the BSEEJ graphical model. In this manner, Algorithm S2 intentionally biases the selection of junctions that have stronger posterior support. This behavior is explained in the methods section of the manuscript; "we use β_k to guide the proposed SEEJs".

After Gibbs sampling converges, BSEEJ collapses SEEJs with the same junction configuration.

Algorithm S2 Local Independent Set Search

Input: β_k, T, S, $G = (V_g, E_g)$, $\omega(\bar{G})$
Output: Φ

$\Phi \leftarrow \emptyset$
while $|\Phi| < T$ **do**
  $r \leftarrow \text{Bernoulli}(1 - \frac{|S|}{\omega(\bar{G})})$ ▷ Sample proportional to $\omega(\bar{G})$
  if $r = 1$ **then**
    $\mathcal{N}_S \leftarrow \{u \in V_g \{u,v\} \in E_g \text{ for some } v \in V_g\}$ ▷ Set S neighborhood
    $free \leftarrow V_g \setminus (\mathcal{N}_S \cup S)$
    if $free \neq \emptyset$ **then**
      $sel \leftarrow \text{Cat}(\beta_{k,i \in free})$ ▷ Among the nodes that if added, keeps S independent set, select based on their β distribution
      $S \leftarrow S \cup \{sel\}$
      $\Phi.append(S)$
    end if
  else
    $del \leftarrow \text{Cat}(1 - \beta_{k,i \in S})$ ▷ Among S, Select based on their β distribution
    $S \leftarrow S \setminus \{del\}$
    $\Phi.append(S)$
  end if
end while

Additional Notes for the Minimum Node Cover Algorithm

To identify the minimum node cover of an interval graph G we implement an incremental algorithm [Marathe et al., 1992]. Leveraging the properties of the interval graph, the algorithm first orders the vertices of the interval graph according to a perfect elimination ordering in time and space linear in $|E_g|$ [Golumbic, 2004, Ramalingam and Rangan, 1988]. Then, at each iteration, the minimum edge index which is adjacent to vertices is obtained. This index captures the nesting property of the maximal clique in the graph. Finally, by updating a weight counter associated to each node according to their index, we add vertices to the minimum node cover if necessary. Since calculating the minimum index for vertices takes linear time and for each vertex, the number of weight updates is equal to the degree of that vertex (overall $\mathcal{O}(\sum_{v \in V_g} d_v)$ where d_v is the degree of vertex v), the time complexity of the algorithm is on the order of $\mathcal{O}(|E_g|)$, where $|E_g|$ is the cardinality of edge set in the interval graph.

Sample Processing

This section provides preprocessing commands used to prepare the simulated and experimental data.

STAR

GEUVADIS BAM files were downloaded from ArrayExpress (accession E-GEUV-6), which were generated by aligning fastq files using TopHat version 2.0.9 and human genome assembly version hg19. We generated the EGA and simulated data BAM files using the STAR aligner (version 2.7.3a). In the example below, ${1} is the directory where the input files are located. The input file 'person_*_1.fa' is a collection of genes for the ith sample on forward sequence and the second is the collection of genes on the backward sequence. This enables using `twopassMode`, which we combined with the `intronMotif` option to obtain spliced alignments. After alignment, the resulting BAM files are partitioned by gene coordinates.

```
STAR --runThreadN 20 --genomeDir ../genome_data/genome_index/ \
     --outFileNamePrefix ./person_${i}_ \
     --twopassMode  Basic --outSAMstrandField intronMotif \
     --outSAMtype BAM SortedByCoordinate \
     --readFilesIn ${1}/person_${i}_1.fa ${1}/person_${i}_2.fa
```

Regtools

Regtools was used for an efficient filtering of the junctions. In the command below, the two %s indicate a BAM file and output filename, respectively.

```
regtools junctions extract -s 0 -a 6 -m 50 -M 500000 %s -o %s.junc
```

On all data used in this project, EGA, Geuvadis, and simulations, we used the following flags:

- -s: finds XS/unstranded flags
- -a: minimum anchor length into exon (6 bp)
- -m: minimum intron size (50 bp)
- -M: Maximum intron size (500000 bp)

Portcullis

Portcullis was run on both simulation and experimental data. The first step of portcullis is preparing the reference genome FASTA file; we present here an example used in the data simulations.

```
portcullis prep -t 20 -v --force -o %s_portcullis/1-prep/ \
      GRCh38.primary_assembly.genome.fa %s/%s.bam
```

Here %s is the name of the output folder and BAM file.

```
portcullis junc -t 20 -v -o %s_portcullis/2-junc/portcullis_all \
      --intron_gff %s_portcullis/1-prep/
```

The next step is to extract junctions in a GFF format. Here %s refers to the name of the folder to search.

```
portcullis filt -t 20 -v -n --max_length 500000 \
      --min_cov 30 -o %s_portcullis/3-filt/portcullis_filtered \
      --intron_gff portcullis_all.junctions.tab
```

Finally, we filter junctions; we retain introns that have a length less than 500000 and have a minimum coverage of 30. Here %s indicates the gene folder to search for the input files. After portcullis is complete, we keep junctions with a 90% overlap between regtools and portcullis.

Additional Results

Details on Differential Expression Analysis.

For the differential expression analysis in synthetic data, we allocated each method a full week on a 128 core computer to processes the simulated data. StringTie, rMATS, LeafCutter, and BSEEJ all finished in less than a day, while Cufflinks only finished 21.4% of the configurations. Similarly, rnaSPAdes required approximately 3 days to finish processing each simulated gene. Additionally, rMATS, StringTie, and LeafCutter generally perform favorably for differential splicing detection when compared to Cufflinks and MAJIQ [Li et al., 2018, Shen et al., 2014, Pertea et al., 2015]; thus, we excluded Cufflinks, MAJIQ, and rnaSPAdes from the comparison.

Node Cover Sizes.

The size of the model depends on the minimum node cover size, which grows as a function of the number of unique intron excisions and their overlaps. We computed the minimum node cover size (and therefore model size) as a function of the number of unique intron excisions for each gene and across all datasets (Fig. S2).

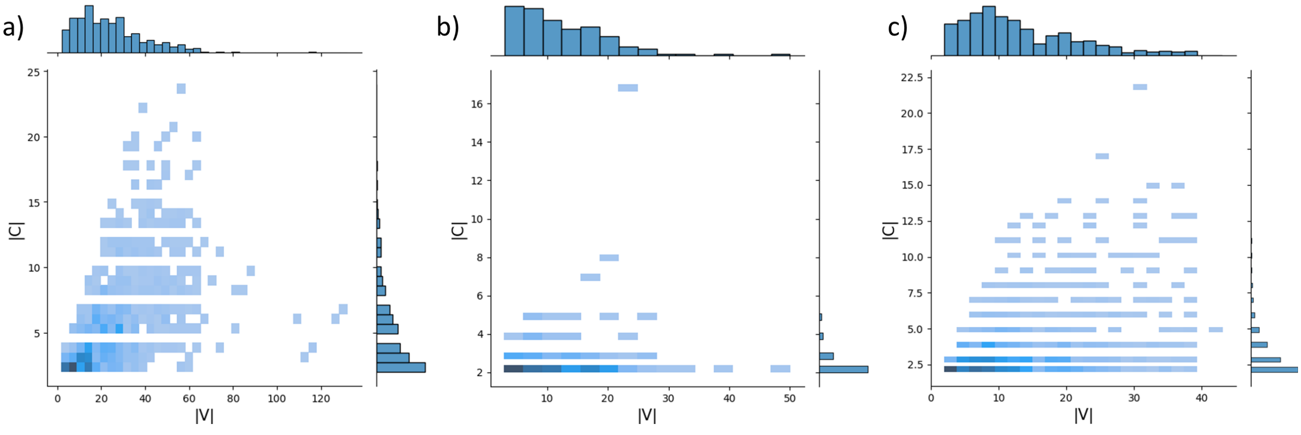

Fig. S2. Model size as a function of the input. A heat map and histograms of the minimum node cover set size with respect to the number of unique intron excisions in a) simulated data b) GEUVADIS data and c) EGA data.

Evaluation of Transcript Segment Matching on the Full Simulated Data

We evaluated the transcript reconstruction performance of all six methods with respect to partial homogeneity scores, for all the genes.

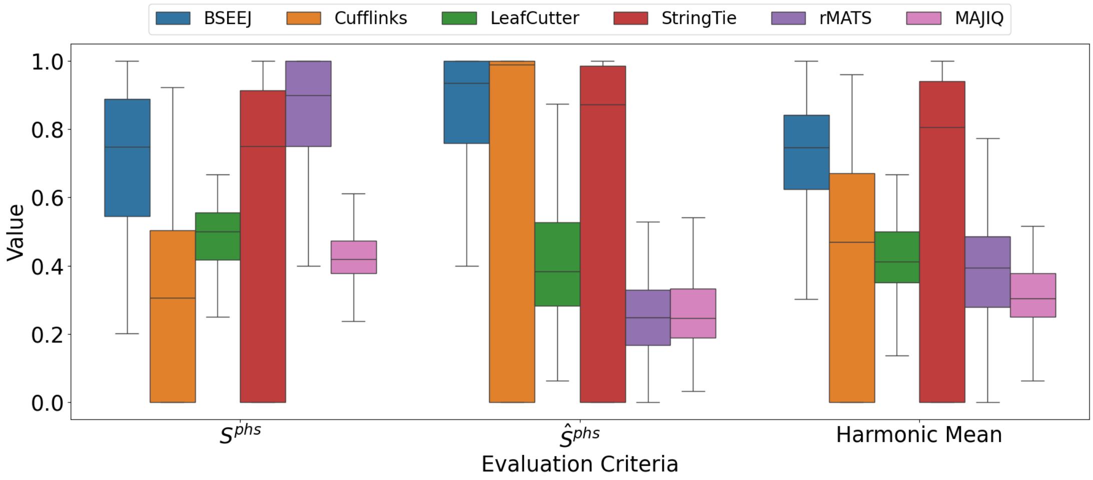

Fig. S3. Transcript segment matching to reference. Box plots for s^{phs}, $\hat{s}^{phs}$ and their harmonic mean across six methods in the full simulated data. Each box shows the median, interquartile range (IQR), and Tukey whiskers ($1.5 * \text{IQR}$ from the nearest hinge).

Chromatic number of genes

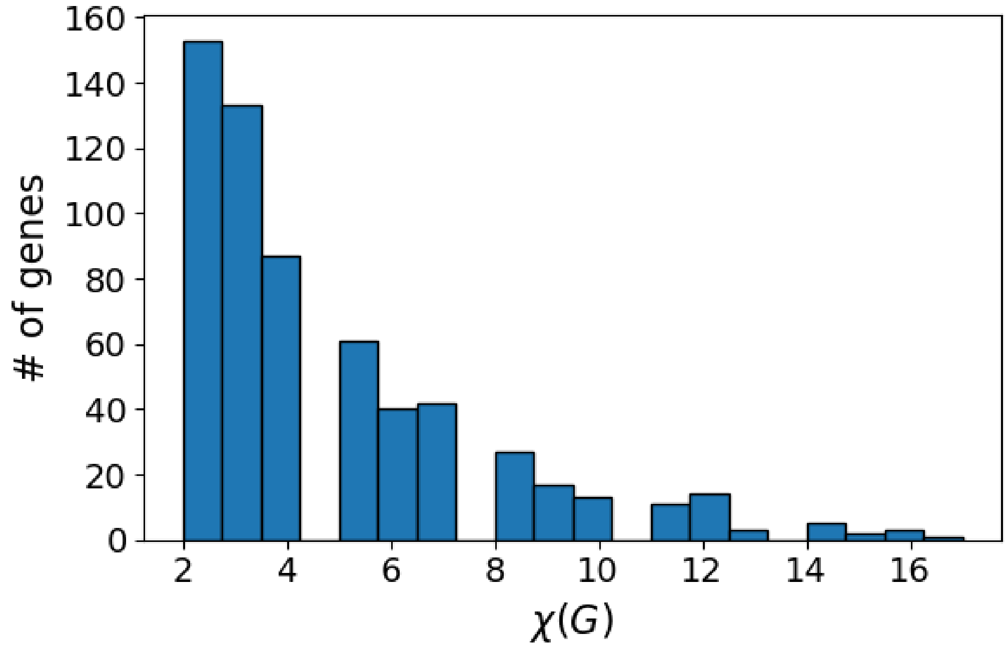

Fig. S4. Histogram showing the distribution of the chromatic number $\chi(G)$ across genes in simulated data.

BSEEJ *Performance as a Function of* $\boldsymbol{K}$.

We evaluated the impact of the parameter that controls the number of SEEJs (K) on the precision and recall; we varied K from the chromatic number in the interval graph (equivalently, the size of the maximum independent set (IS) in the complement interval graph) to $IS + 16$. Fig. S5 shows precision and recall for s^{phs} (top) and $\hat{s}^{phs}$ (bottom).

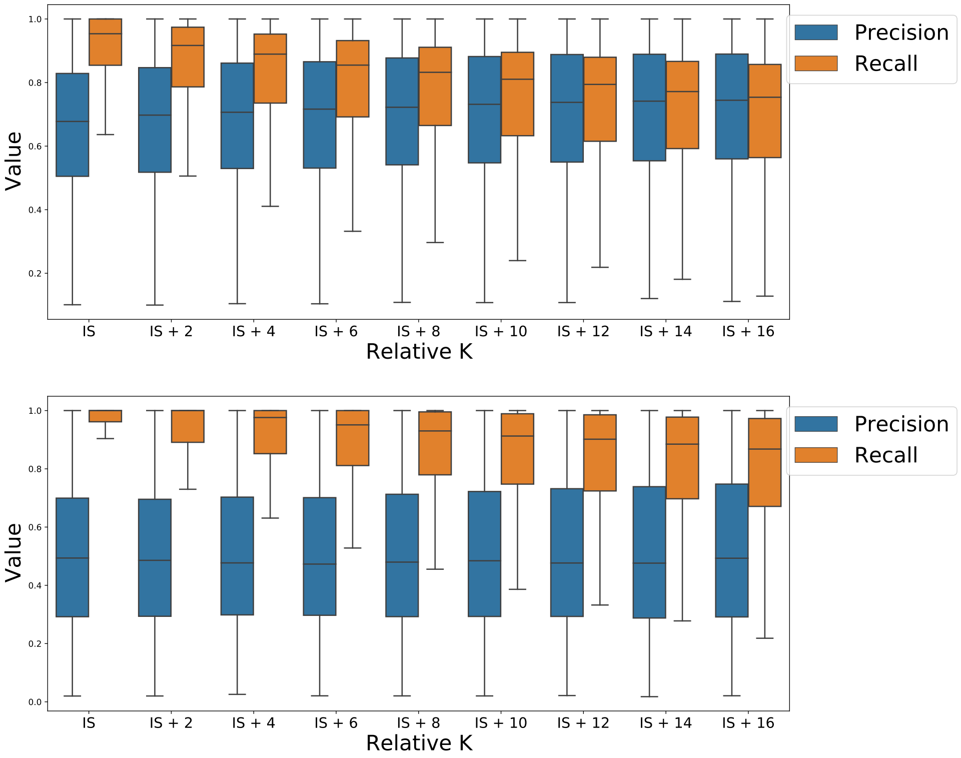

Fig. S5. Precision and recall for s^{phs} (top) and $\hat{s}^{phs}$ (bottom) in models where $K = IS + \{2, 4, 6, 8, 10, 12, 14, 16\}$ and IS is the size of maximum independent set in the complement of the interval graph.

Evaluation of Transcript Reconstruction on the Full Simulated Data

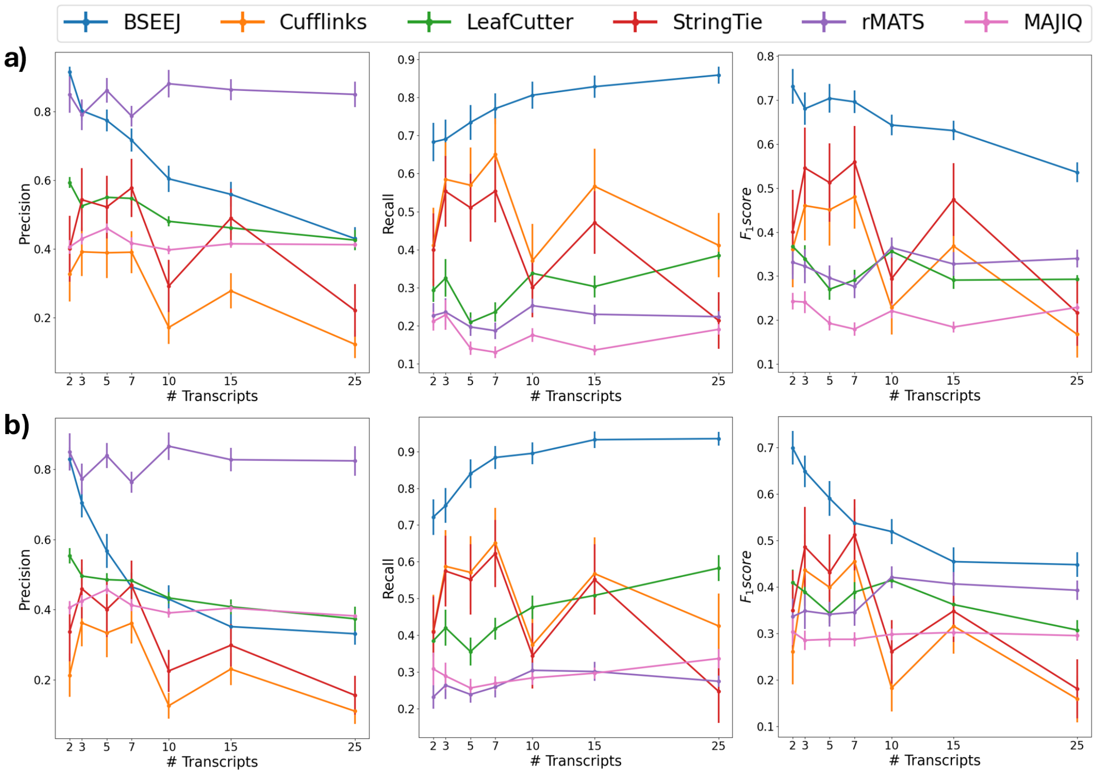

Fig. S6. Transcript reconstruction performance on simulated data. Precision, recall and F_1 score on simulated data for BSEEJ (blue), Cufflinks (orange), LeafCutter (green), StringTie (red), rMATS (purple), and MAJIQ (pink) based on **(a)** S^{phs} and **(b)** $\hat{S}^{phs}$ for the full simulated data.

Evaluation of Transcript Reconstruction with Extended Metrics

In addition to precision and recall, we computed weighted harmonic means of the them, to emphasize different trade-offs. Specifically, $F_{0.5}$ places more weight on precision, while F_2 places more weight on recall, and F_1 balances them equally. These complementary metrics allow a more nuance comparison of the method behavior.

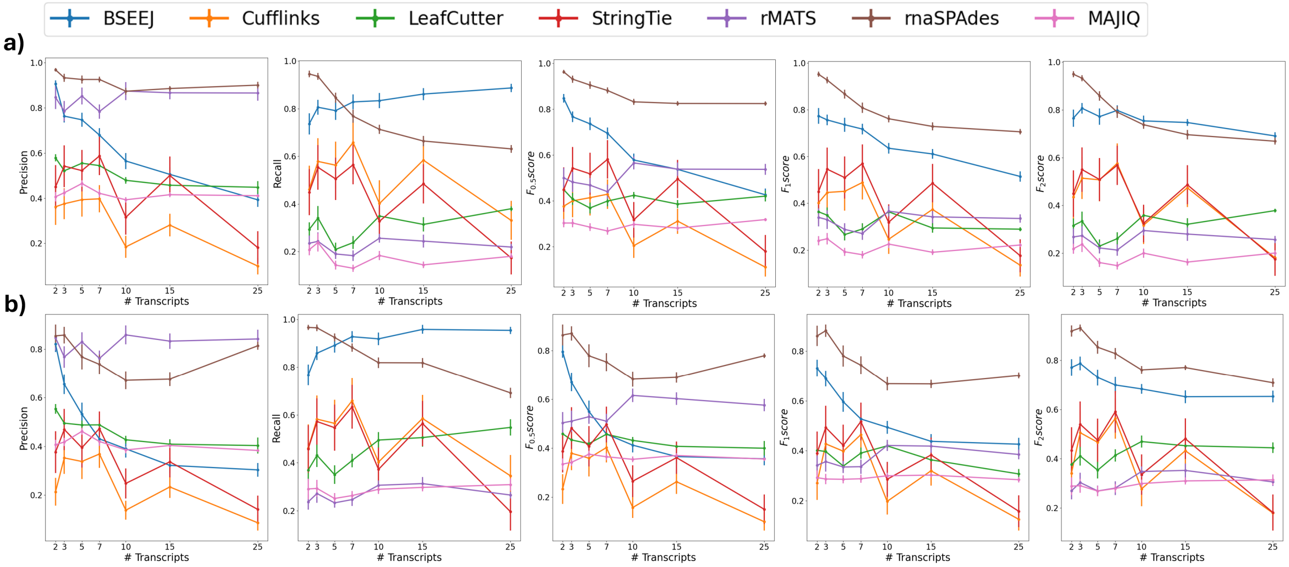

Fig. S7. Transcript reconstruction performance on simulated data. Precision, recall, F_β ($\beta \in \{0.5, 1, 2\}$) score on simulated data for BSEEJ (blue), Cufflinks (orange), LeafCutter (green), StringTie (red), rMATS (purple), rnaSPAdes (brown), and MAJIQ (pink) based on **(a)** S^{phs} and **(b)** $\hat{S}^{phs}$ for the full simulated data.

Performance on Complex Genes.

We evaluated transcript segment reconstruction for genes that have a substantial overlap in the interval graph; that is, we considered genes where the number of junction overlaps exceed 200 ($|E_g| > 200$; Fig. S8).

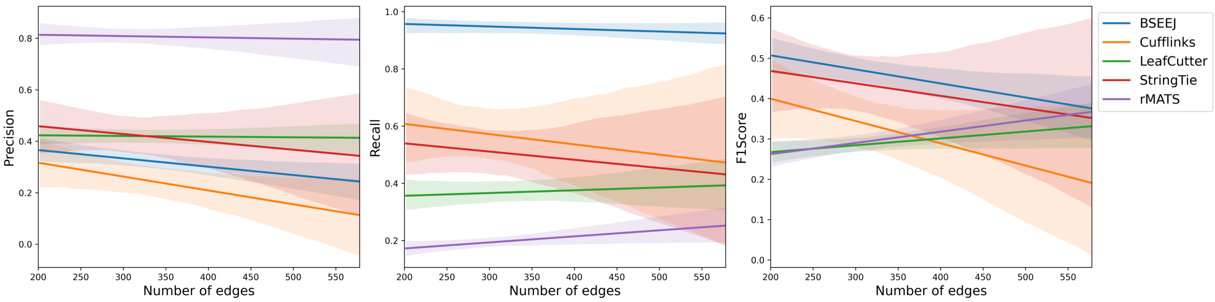

Fig. S8. Performance in complex genes where the number of edges exceed 200. The x-axis shows the number of edges in the interval graph and the y-axis is the performance metric.

We also considered performance as a function of the number of junctions (not necessarily with high junction overlap; Fig. S9).

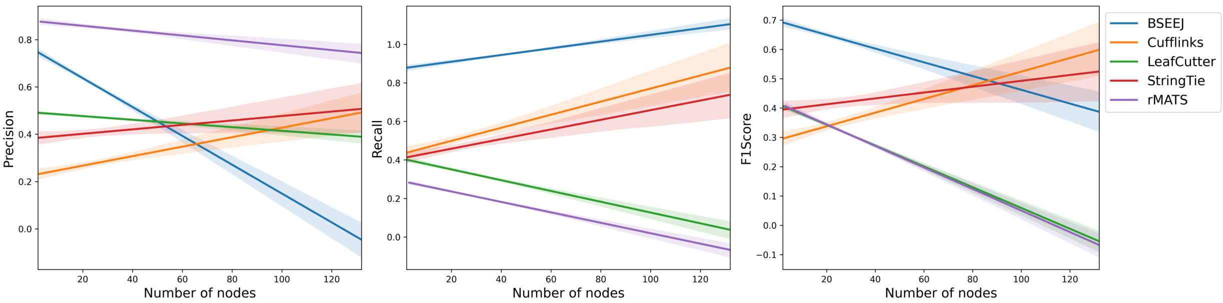

Fig. S9. Performance as a function of number of junctions. The x-axis shows the number of nodes in the interval graph (unique junctions) and the y-axis is the performance metric.

Sensitivity Analysis 1

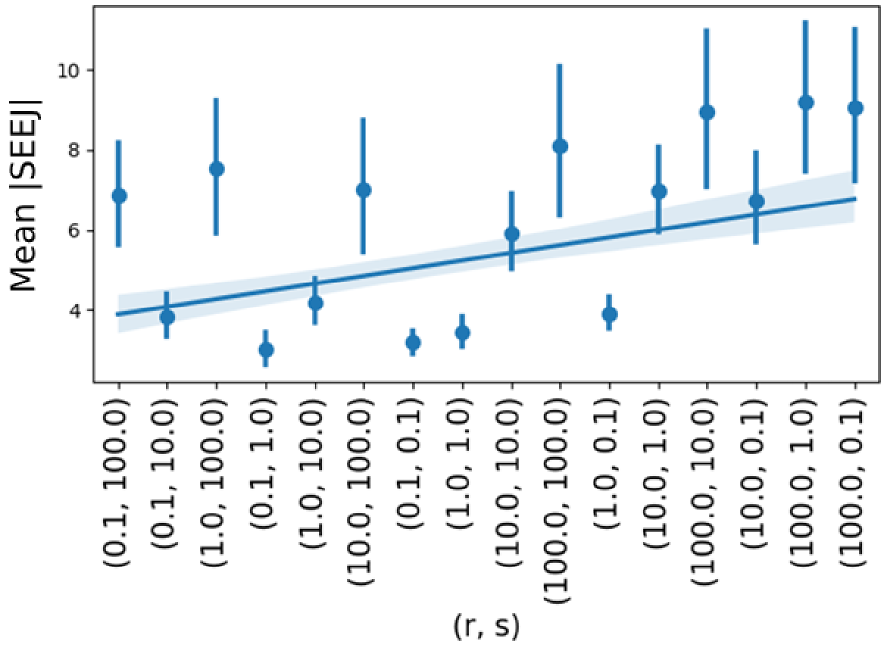

Fig. S10. Sensitivity analysis of SEEJ size with respect to the parameters r and s. The x-axis depicts combinations of the π prior hyperparameters, ordered by their mean, i.e., $\frac{r}{r+s}$. The y-axis represents the average SEEJ size with bars indicating 95% confidence intervals.

BSEEJ *Runtime.*

Since the number of iterations varied from different runs of the same gene, we computed the runtime of BSEEJ per iteration for the simulated groups of genes. We omitted the group of 17 genes that had an average number of junction reads that was larger than 60,000 since this group was small relative to the size of the simulation (1260 genes). Then we plotted the run time as a function of K, the size of minimum node cover, and the average number of junction reads (Fig. S11). The model was trained on a server with Intel(R) Xeon(R) Gold 6242 @ 2.80GHz CPUs.

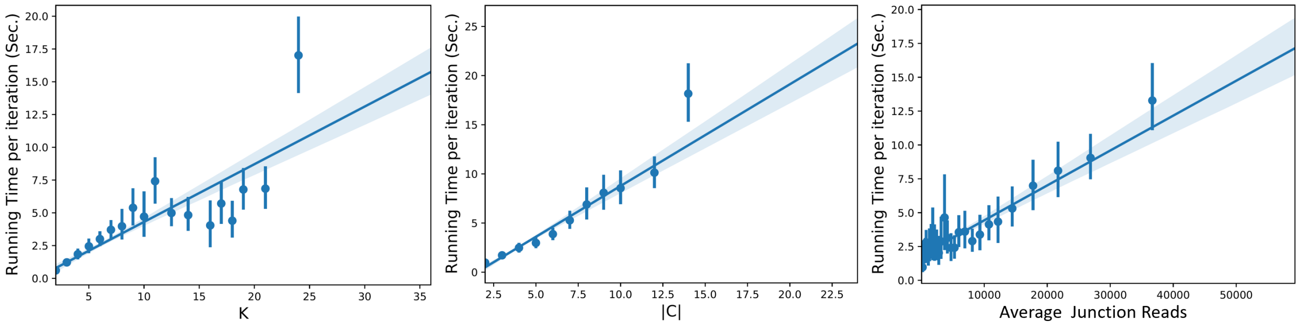

Fig. S11. BSEEJ runtime (sec. per iteration) as a function of (left) the prior number of SEEJs (K), (middle) the size of the minimum node cover set, (right) the average number of junction reads across samples.

Runtime of reconstruction methods on simulated data.

To evaluate computational runtime of the methods, we randomly selected 10 genes with 5× coverage and compared the total runtime until convergence for BSEEJ to other methods' total runtime (Fig S12).

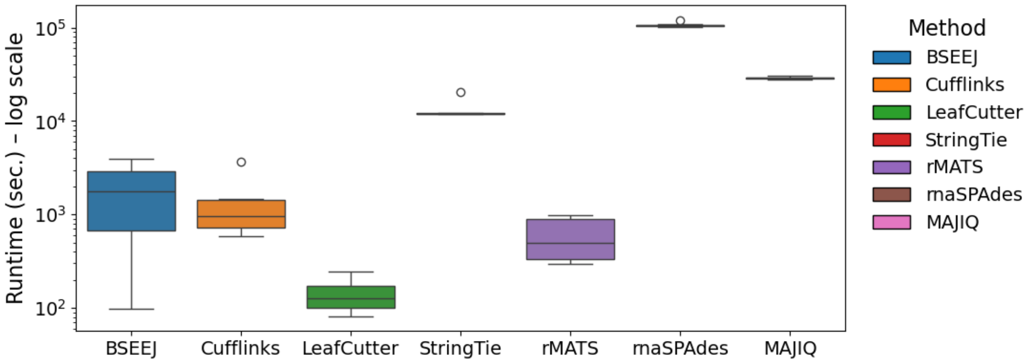

Fig. S12. Runtime of methods. Computational runtime (seconds, log scale) for all evaluated methods (BSEEJ, Cufflinks, LeafCutter, rMATS, rnaSPAdes, MAJIQ) on a subset of genes. Each box shows median runtime, interquartile ranges (IQR), whiskers (1.5 ∗ IQR from the nearest hinge), and outliers.

P-value Calibration.

We plotted the distribution of p-values from our differential SEEJ analysis in the GEUVADIS and EGA data (Fig. S13).

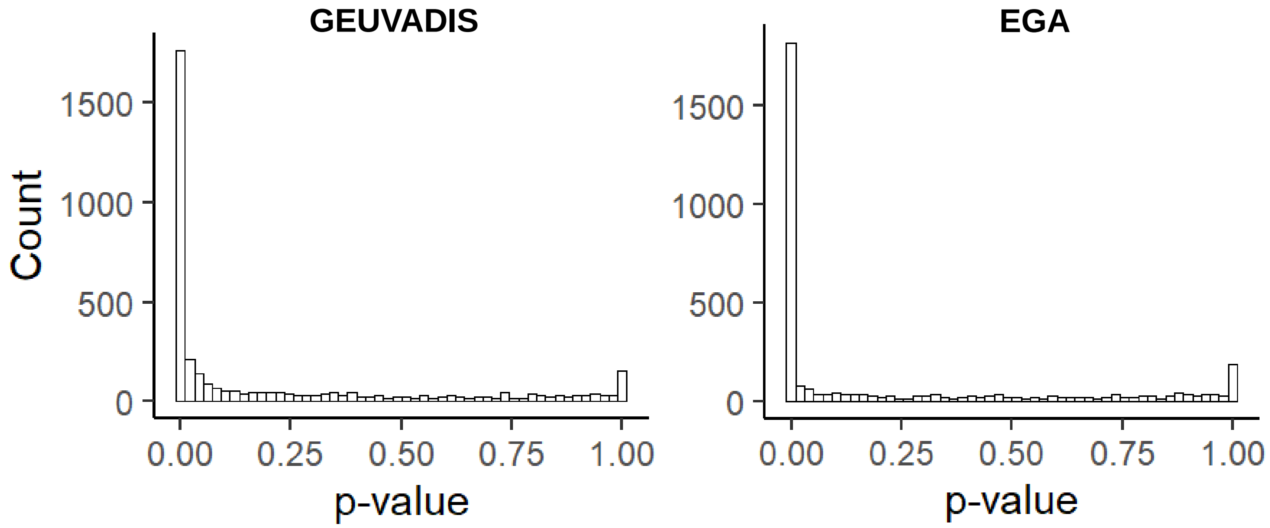

Fig. S13. Differential SEEJs are significantly enriched in the GEUVADIS and EGA data.

Quantitative results on experimental data

We computed quantitative metrics (precision, recall, F_1-score) for evaluating the quality of reconstructed SEEJs produced by BSEEJ in the GEUVADIS and EGA datasets. We used the GENCODE v19 annotation (GRCh37/hg19) as a gold-standard reference annotation, consistent with the genome build originally used by GEUVADIS and EGA studies. The junction coordinates from the reconstructed SEEJs were compared directly against annotated exon-exon junctions from GENCODE. We defined true positives as predicted SEEJs where the start and end positions of each junction matched the corresponding junction boundaries in the GENCODE reference annotation within 6 nucleotides following LeafCutter[Li et al., 2018]. False positives and false negatives were defined analogously.

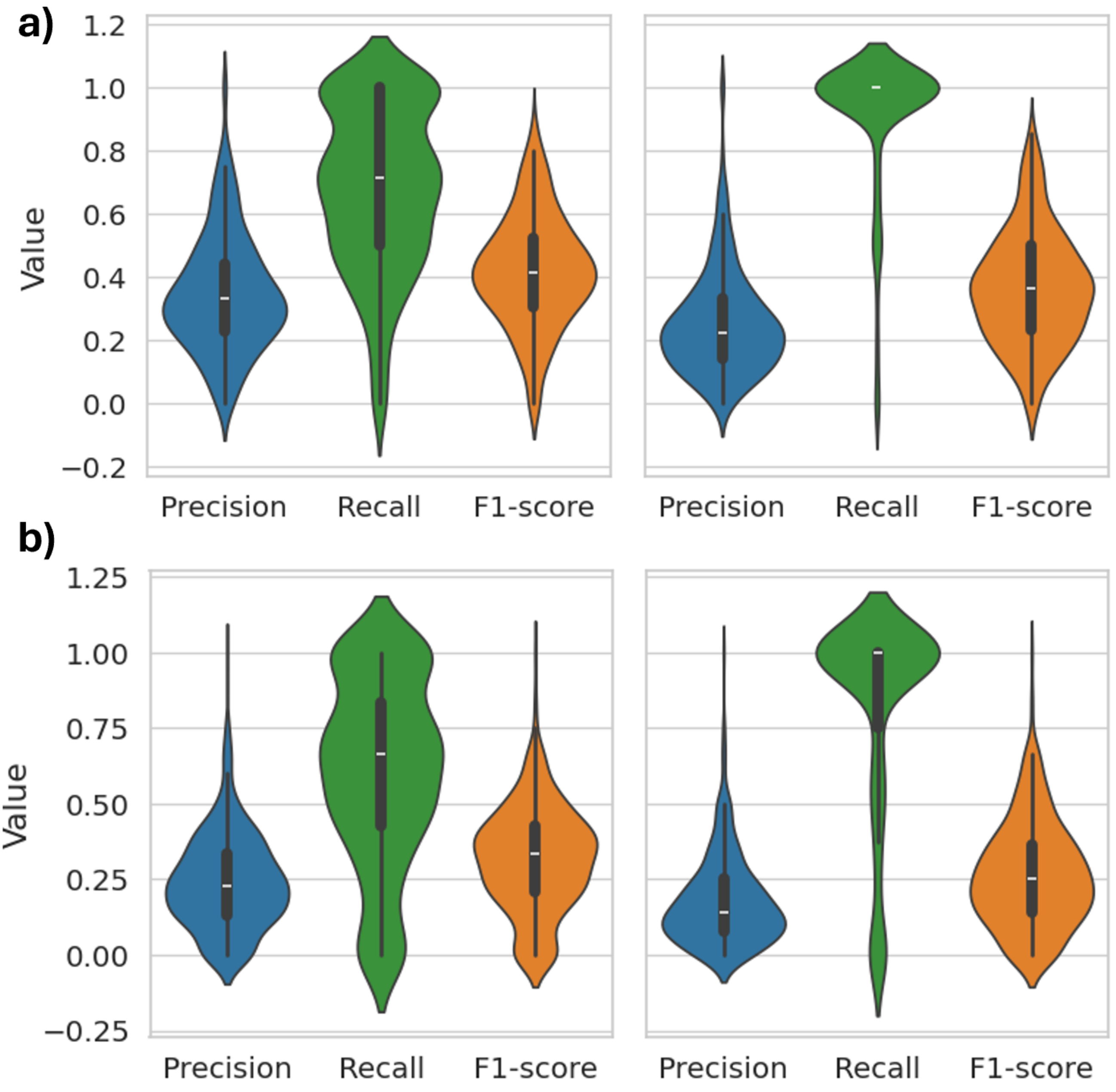

Fig. S14. Distribution of BSEEJ performance metrics on the experimental data. Each violin shows the empirical distribution of one evaluation metric across the genes for a) GEUVADIS and b) EGA. The embedded box indicates the inter-quartile range. The metrics in the left and right panels are computed based on S^{phs} and $\hat{S}^{phs}$, respectively.

Novel Introns and SEEJs in Experimental data

We computed the count and percentage of introns and SEEJs that are present in or absent from the annotation reference file. Since our processing pipeline focuses on junctions, and is thus similar to LeafCutter, and we are testing reconstruction, we compared our results to only Cufflinks and StringTie. After running the methods on experimental genes, we merged single individual transcript reconstructions to produce a single file per gene for Cufflinks and StringTie. A SEEJ is expressed if it contains more than 10 junctions mapped to it. Additionally, the number of samples that express that SEEJ must be larger than 10. Then in the merged file per gene, if a SEEJ in BSEEJ or a transcript in StringTie and Cufflinks is a subset of any of the annotated transcripts, we count it as present, otherwise it is counted as absent (Fig. S15). For the percentage plots, we compute the percentage of present or absent for splice junctions and SEEJs or transcripts separately (Fig. 5).

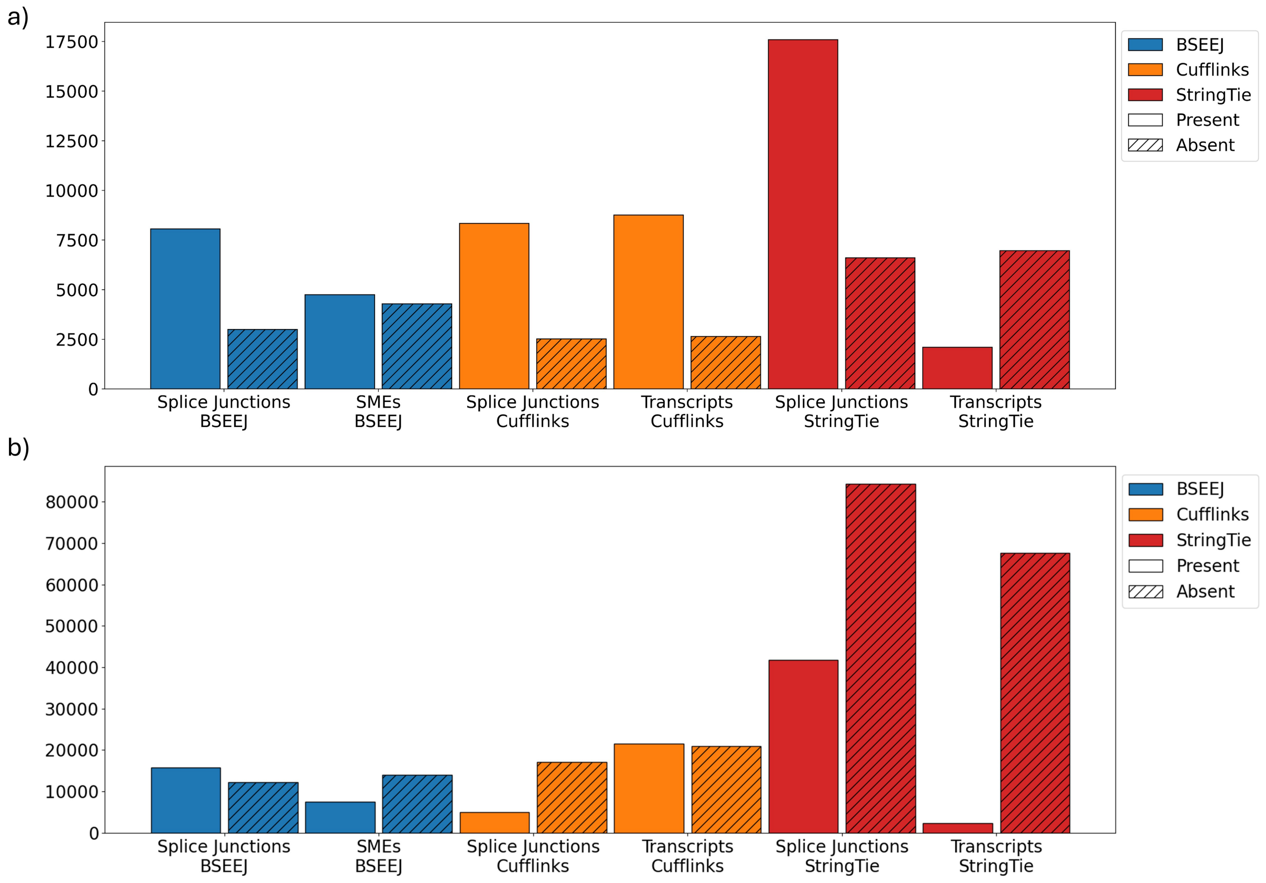

Fig. S15. The count of mRNA junctions and SEEJs or transcripts that are present or absent in the reference annotations across BSEEJ, Cufflinks, and StringTie for experimental data. The two plots show the counts of present and absent junctions for SEEJs or transcripts in a) GEUVADIS and b) EGA.

Performance as a function of the number of samples

We investigated the association between BSEEJ F_1 score and the number of samples per gene. In our experimental datasets, we averaged F_1 scores across all genes for each sample count and computed non-parametric rank-correlations Spearman and Kendall. For the Geuvadis dataset, the Spearman and Kendall correlations were −0.091 and −0.058; for the EGA dataset, the Spearman and Kendall correlations were 0.026 and 0.014.

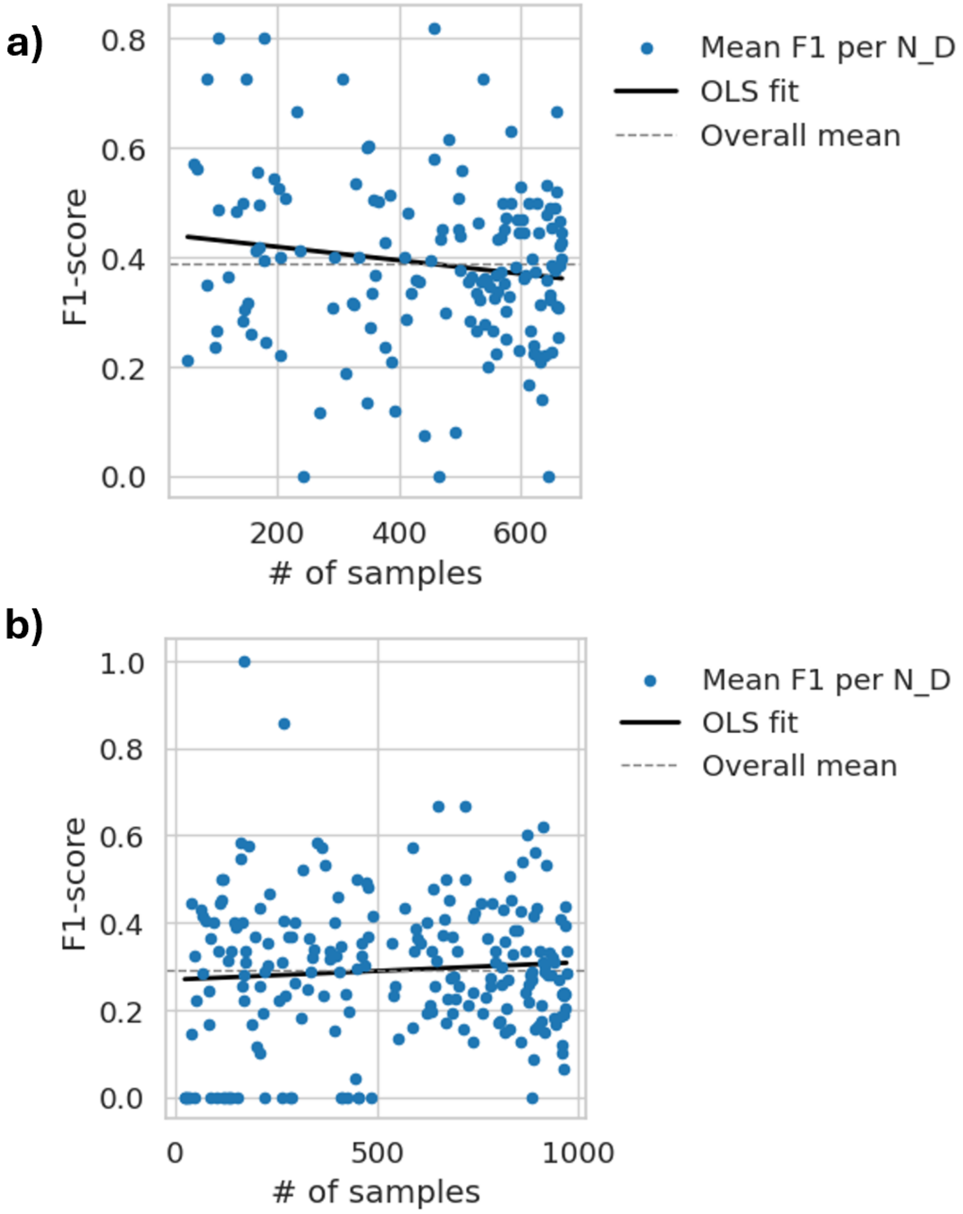

Fig. S16. The association between F_1 score and the number of samples per gene. Two plots show the fitted relationship between the mean per-gene F_1 score and the number of samples in a) GEUVADIS and b) EGA datasets. Each blue dot represents the average F_1 score of all genes at a particular sample count; the solid black line is the ordinary-least-squares (OLS) fit to those points.)
